# Seasonal variation in tap water δ^2^H and δ^18^O isotopes reveals two tap water worlds

**DOI:** 10.1038/s41598-020-70317-2

**Published:** 2020-08-11

**Authors:** Ruan F. de Wet, Adam G. West, Chris Harris

**Affiliations:** 1 Biological Sciences Department, University of Cape Town, Rondebosch, 7701 South Africa; 2 Geology Department, University of Cape Town, Rondebosch, 7701 South Africa

**Keywords:** Hydrology, Environmental monitoring, Geochemistry

## Abstract

Stable isotope ratios of hydrogen and oxygen (δ^2^H and δ^18^O) in tap water provide important insights into the way that people interact with and manage the hydrological cycle. Understanding how these interactions vary through space and time allows for the management of these resources to be improved, and for isotope data to be useful in other disciplines. The seasonal variation of δ^2^H and δ^18^O in tap water within South Africa was assessed to identify municipalities that are supplied by seasonally invariant sources that have long residence periods, such as groundwater, and those supplied by sources that vary seasonally in a manner consistent with evapoconcentration, such as surface water—the proposed two tap water “worlds”. Doing so allows for the cost-effective spatial interpolation of δ^2^H and δ^18^O values that likely reflect that of groundwater, removing the residual error introduced by other sources that are dependent on discrete, isolated factors that cannot be spatially generalised. Applying the proposed disaggregation may also allow for the efficient identification of municipalities that are dependent on highly variable or depleted surface water resources, which are more likely to be vulnerable to climate and demographic changes.

## Introduction

Understanding the variation of stable hydrogen (H) and oxygen (O) isotopes in tap water has global application for several diverse fields^1^. In many contexts, the management of water resources is expected to become increasingly challenging given the predicted impacts of climate change, urbanization, economic development, and increasingly complex water resource supply systems and infrastructure^2,3^.

**Figure 1:**
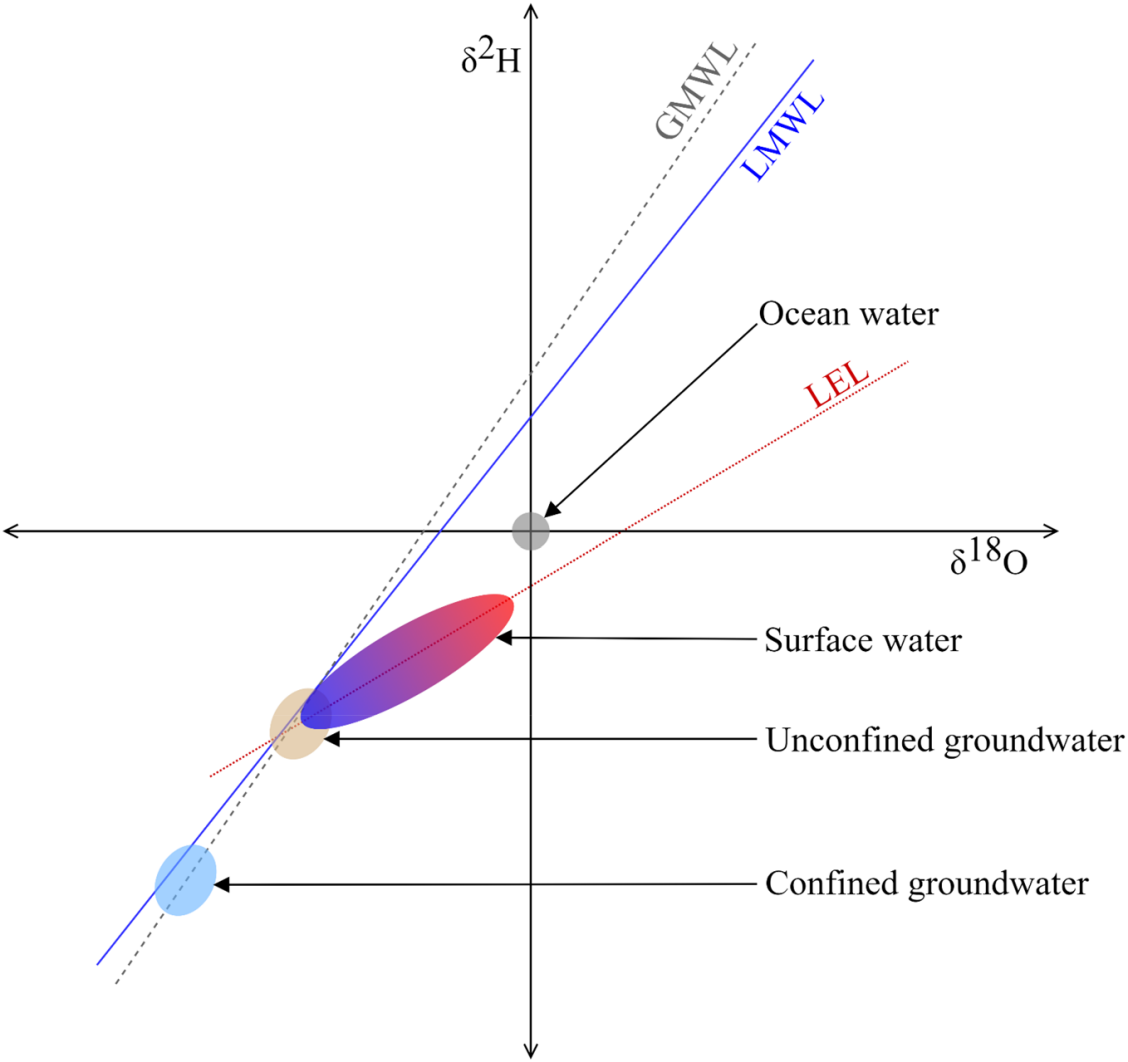
Hypothetical δ^2^H and δ^18^O values for surface water, confined and unconfined groundwater, and ocean water relative to the global and local meteoric water lines (GMWL and LMWL), and local evaporation line (LEL). Surface water varies along the LEL due to evapoconcentration in the dry months and recharge in the wet months. Groundwater is comparatively invariant with isotopes in unconfined groundwater reflecting that of mean annual precipitation and confined groundwater differing from the local mean annual precipitation but rather reflecting that of the spatially or temporally distant recharge source (such as paleorecharge). The δ^2^H and δ^18^O values in ocean water, by definition, approximate the origin. See Supplementary Information for further details.

Municipal tap water is a primary interface between people and the hydrological system. Most municipalities are supplied by some combination of local surface and groundwater but may also rely on non-local sources through inter-basin water transfer schemes and processed water such as treated sewage effluent or desalinated seawater^4,5^. A theoretical understanding of the seasonal variability of stable isotopes in precipitation, surface water and groundwater (Fig. 1) provides the foundation from which informed inferences can be made about the seasonal variability of stable isotopes in tap water. Some of these inferences have recently been corroborated^2,6–13^, but others remain untested.

Understanding the source of tap water supply is important for sustainable water resource management, as the associated risks, including those due to climate change, differ between sources^10^. Greater short-term variability in water input from precipitation, for example, would have a greater impact on hydrological systems that have a limited storage capacity, such as smaller surface water reservoirs, than those with longer residence times, such as groundwater aquifers or large surface water reservoirs. Furthermore, the monitoring of groundwater resources is critical to the sustainability of water supply, especially where groundwater resources are relied upon to buffer the effects of greater climate variability and where water demand has exceeded the supply capacity of traditional surface water resources. Information on the source of municipal tap water is not always available, reliable or independently verifiable, particularly in low- and middle-income countries. The available groundwater information in South Africa, which is one of the most data-rich countries in Southern Africa, for example, is inadequate for management purposes^14^ and reliable records are only available for fewer than a third of the major surface water reservoirs^15^. Stable isotopes in tap water offer an approach to monitoring the dynamics and utilisation of critical water resources at a national scale.

The primary aim of this study is to test a cost-effective method of identifying and quantitatively characterizing the two tap water worlds—the first, groundwater with seasonally invariant δ^2^H and δ^18^O values, the second, surface water with seasonally variant δ^2^H and δ^18^O values—using stable isotopes in tap water. This method was tested at a national scale using tap water from South Africa as a case study. Successful disaggregation between groundwater and surface water sources would provide greater insight into the dynamics of the water supply system. It would enable the identification of municipalities that may be vulnerable to predicted climate and demographic changes, enable the prioritisation of adaptation interventions to increase the resilience of the water supply system, and it would provide a framework to directly and cost-effectively monitor the water resources on which municipalities are reliant.

## Methods

### Collection of samples

#### Return mail campaign

Two national return-mail sampling campaigns were undertaken across South Africa for this study, in May and November 2017, representing the end of the summer and winter seasons respectively. Sample kits were sent to 340 branch managers of the South African Post Office following the methods of West et al.^8^. Intact, viable sample kits were returned in either the May or November campaign by 278 (82%) post office branches, with 233 (69%), 203 (60%) and 158 (46%) branches returning viable sample kits in May, November and both campaigns, respectively (Table S1).

### Analysis of samples

#### Isotope analysis

Stable water isotopes were analysed at the University of Cape Town by wavelength-scanned cavity ring-down spectroscopy using a Picarro L2120-i (Picarro Inc., 480 Oakmean Parkway, Sunnyvale, California, 94085, USA; https://www.picarro.com/) following the methods of West et al.^8^. Prior to analysis, each sample was filtered through a 0.2 µm syringe filter. Six injections of 1.9 µl were analysed for each sample, with the results of the first three injections discarded as conditioners. All samples were assessed for additional components that could affect the analysis following the methods of West et al.^8^. Isotopic ratios are expressed in ‰ as:

$$\delta^{N} E = \left( {\frac{{R_{sample} }}{{R_{standards} }} - 1} \right) \times 1,000$$

where N is the atomic mass of the heavy isotope of element, and E and R is the ratio of the heavy to light isotope (^2^H/H or ^18^O/^16^O). The d-excess is defined as^16^:

$${\text{d } - \text{ excess}} = \delta^{2} H - 8 \times \delta^{18} O$$

For drift correction and quality control, three in-house standards of known δ^2^H and δ^18^O values (1.6 and − 0.6; − 71.7 and − 10.2; and − 129.5 and − 17.27, respectively) where analysed after at most six consecutive samples were run. Tap water samples were calibrated to VSMOW using two in-house standards. The accuracy and precision of the calibration were assessed with a third quality control standard not used in the calibration. The mean (± one standard deviation) accuracy and precision of the isotope analyses across all batches were 0.4‰ ± 0.2‰ and 0.5‰ ± 0.2‰ for δ^2^H and 0.10‰ ± 0.05‰ and 0.09‰ ± 0.05‰ for δ^18^O, respectively.

#### Spatial modelling

A Monte Carlo simulation permutation test for Moran’s I statistic was calculated using 5,000 random permutations for the observed δ^2^H, δ^18^O and d-excess values in R version 3.6.3 (https://cran.r-project.org/)^17^ using the *dismo*^18^ and *spdep*^19^ packages. Universal kriging models were applied to interpolate the observed δ^2^H, δ^18^O and d-excess values and construct prediction isoscapes for each. The additional explanatory variables that were considered include the mean annual precipitation (MAP) and temperature (MAT), elevation (elev) and A-pan equivalent potential evaporation (PE), obtained from Schulze et al.^20^. The shortest Euclidean distance to the coast (tocoast) was calculated in R^17^ using the *rgdal*^21^ and *sf*^22^ packages. Lastly, the predicted mean annual δ^2^H (MAH), δ^18^O (MAO) and d-excess (MAD) in precipitation^23–26^ were also considered.

A linear model was constructed for each isotope ratio from the November and May tap water sampling campaigns using all five climatological and topographical variables (i.e., MAP, MAT, elev, apan and tocoast), the interaction between elev and tocoast (elev*tocoast) and between MAP and apan (MAP*apan), and the corresponding isotope ratio in precipitation (i.e., where δ^2^H in tap water is being predicted, MAH is considered as an explanatory variable but not MAO or MAD). The explanatory variables for the final model were selected by first assessing the stepwise effect on the AIC (Akaike information criterion) value and then the statistical significance of the variable coefficients. The kriging models were run in R^17^ using the *automap*^27^, *gstat*^28^, *sp*^29^, and *raster*^30^ packages, the and plotted using the *tmap*^31^ package. Where the model uncertainty was greater than one standard deviation of the underlying training data, hatching was overlaid on the isoscapes to represent a lower confidence in the model predictions.

#### Quantification and interrogation of seasonal variability

Spatial modelling includes a degree of uncertainty and residual error in its predictions. To investigate the seasonal isotope variation mechanisms, therefore, further interrogation of the data was run on the observed isotope ratios for the 158 locations that provided samples for both campaigns rather than on the interpolated isoscapes that were predicted based on universal kriging.

**Figure 2:**
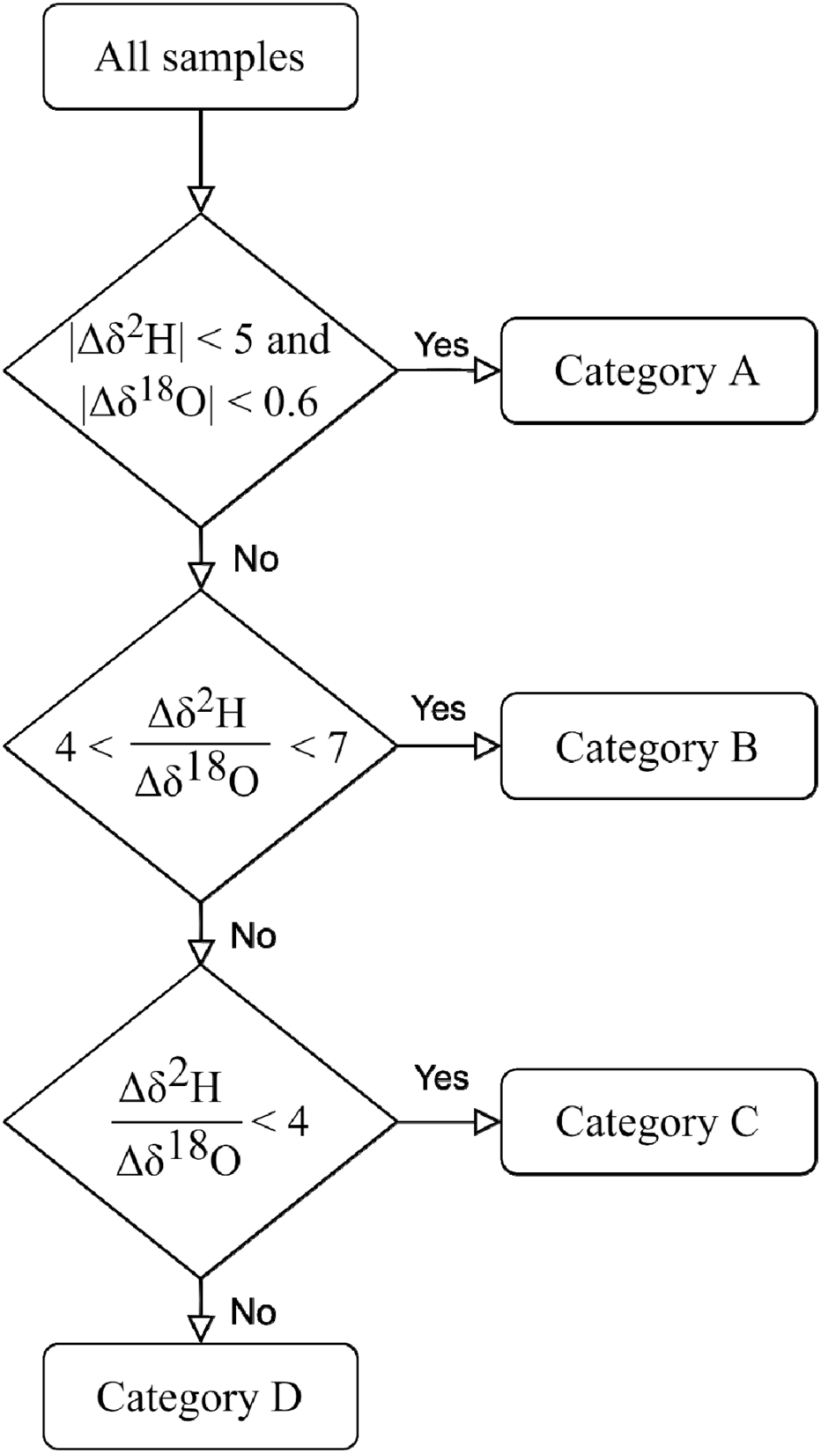
Algorithm applied to categorize the seasonal variability of repeated tap water sampling locations.

The seasonal variability in δ^2^H and δ^18^O of repeated sampling locations was classified based on the magnitude and gradient of variation (Δ) between May and November (Fig. 2) conservatively based on the characterization of surface water δ^2^H–δ^18^O evaporation slopes by Gibson et al.^32^. The thresholds applicable for a given locality differ based on numerous geographical, topographical and climatological factors^33^. The criteria applied in this study are therefore not globally generalisable and warrant further investigation. To allow for any variability resulting from sampling and observational errors^34,35^, a greater margin of variation was allowed for in the classification criteria than would be expected based solely on theoretical principles^33^. The classifications included seasonally invariant locations (Category A; |Δδ^2^H| < 5 and |Δδ^18^O| < 0.6), those that varied in a manner consistent with evaporation (Category B; 4 < $\frac{{\Delta \delta^{2} H}}{{\Delta \delta^{18} O}}$ <7), those that had an atypically shallow gradient of variation (Category C; $\frac{{\Delta \delta^{2} H}}{{\Delta \delta^{18} O}}$ < 4) and those that had an atypically steep gradient of variation (Category D; 7 < $\frac{{\Delta \delta^{2} H}}{{\Delta \delta^{18} O}}$). Each category was further distinguished by the direction of variation between May and November. Sample locations with isotope ratios in November that are more positive than in May are categorized as having a positive direction of variation (i.e., B_p_, C_p_ and D_p_) and vice versa for sample locations categorized as having a negative direction of variation (i.e., B_n_, C_n_ and D_n_).

## Results

**Figure 3:**
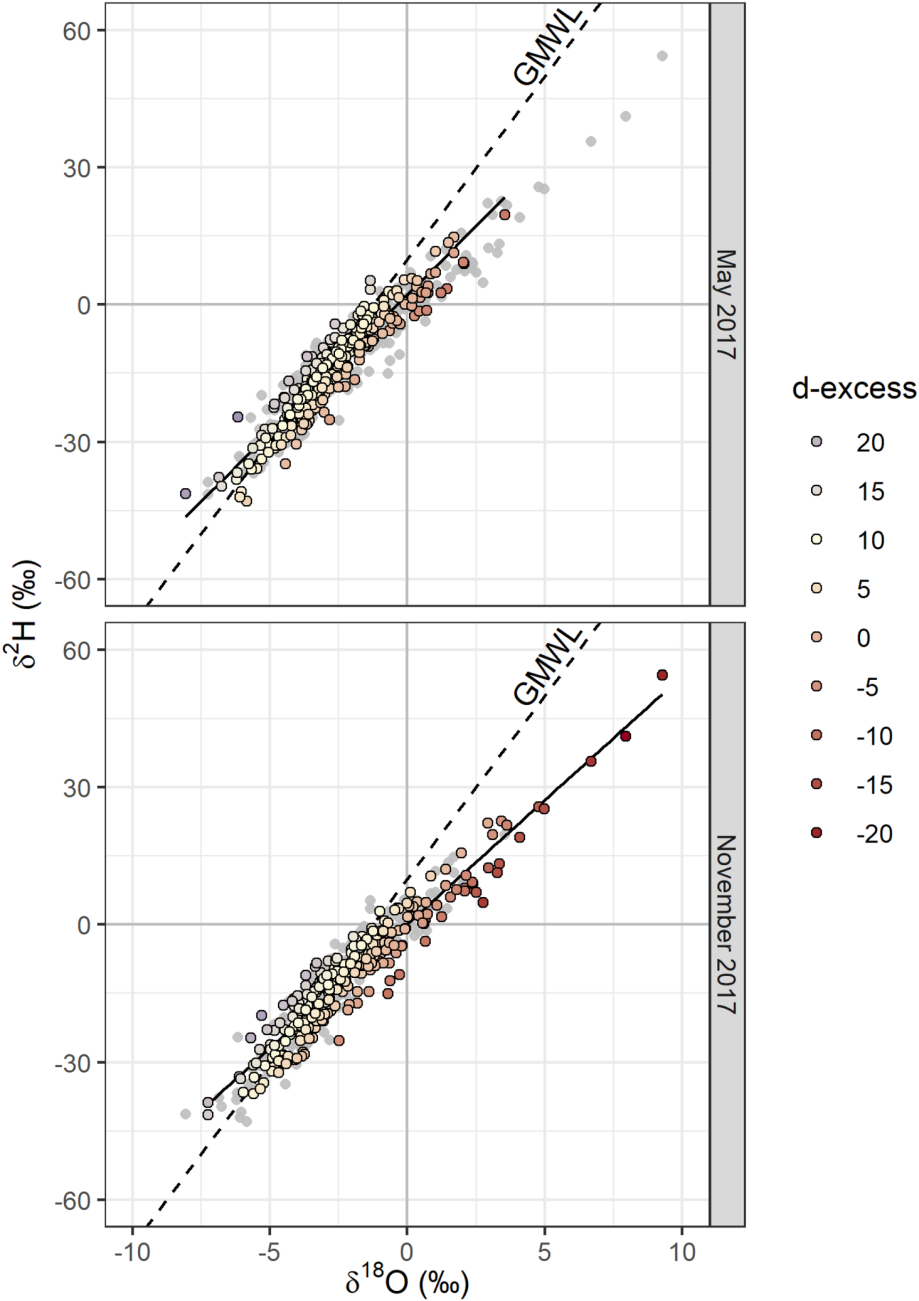
Tap water δ^2^H and δ^18^O values for May (top) and November (bottom) 2017 sampling campaigns. The data for both sampling campaigns are plotted in grey in each panel. For the labelled campaign, deuterium-excess (d-excess) is represented by the colour of each point. The linear regression equation through δ^2^H and δ^18^O space is δ^2^H = 6.0 δ^18^O + 2.3 for May and δ^2^H = 5.4 δ^18^O + 0.1 for November 2017 with an R^2^ of 0.89 and 0.91, respectively.

**Figure 4:**
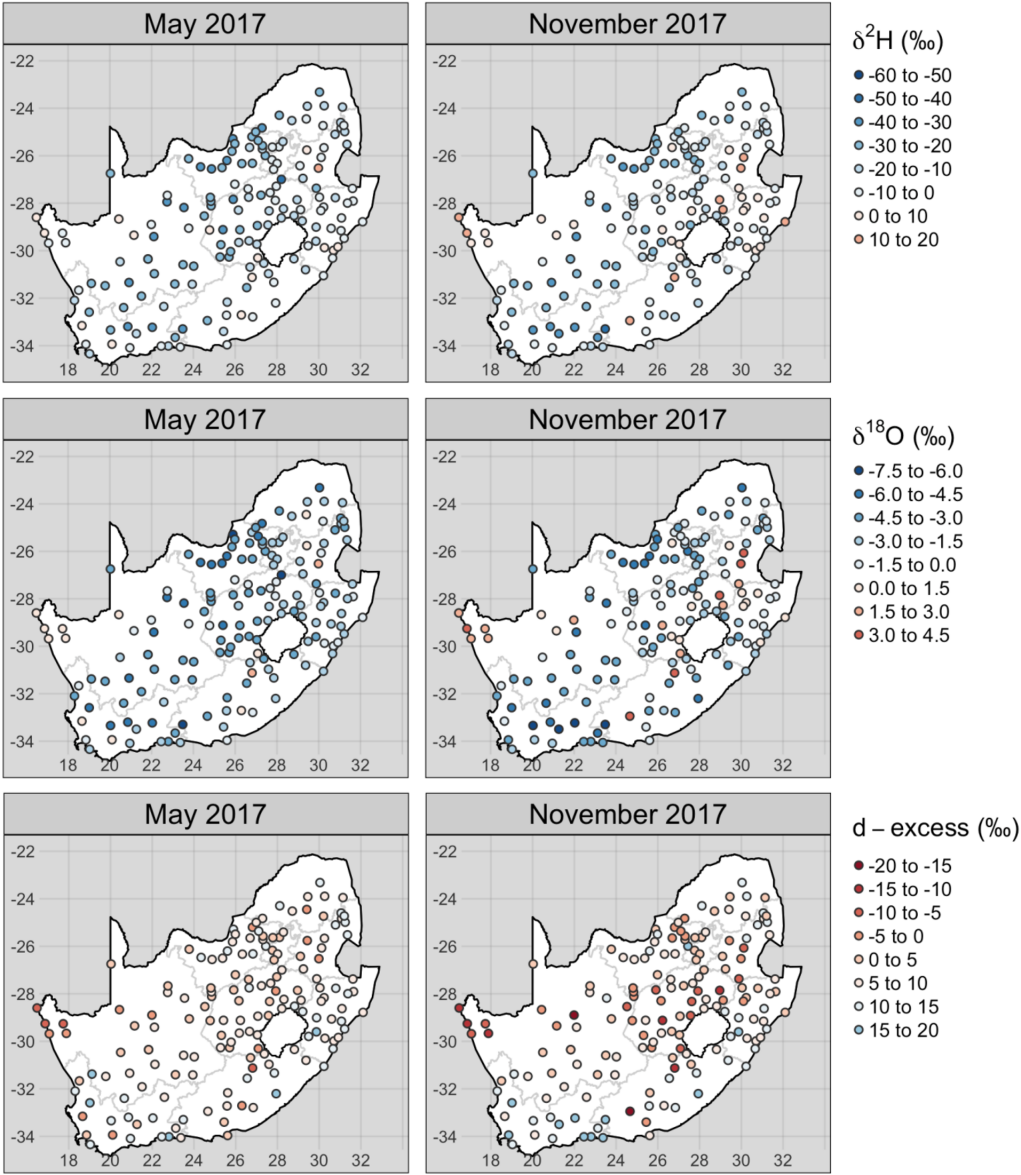
Location of the δ^2^H (top), δ^18^O (middle) and d-excess (bottom) values from the repeated May (left) and November (right) 2017 sampling campaigns.

The overall range (Fig. 3) of δ^2^H, δ^18^O and d-excess November 2017 tap water (− 41.4‰–54.4‰, − 7.26‰–9.27‰$\permil$ and − 22.4‰–22.6‰, respectively) was greater than that of May 2017 (− 43.0‰–19.6‰, − 6.87‰–3.5‰ and − 8.7‰–17.9‰, respectively). There was evidence of greater evapoconcentration in some November 2017 tap water samples relative to that of May 2017, as shown by the number of samples with positive δ^2^H and δ^18^O and negative d-excess values (Fig. 4).

The regional drift in the modelled isotope ratios is best explained by climatic and topographical variables, depending on the isotope ratio and the sampling period (Table 1). The difference in the AIC values of the full and final models was relatively small but, to prevent over-fitting, variables that increased the model AIC were still removed. The highest R^2^ value of the final models was 0.33, with p < 0.001, so a statistically significant amount of the null deviance was described in each case by the explanatory variables. The results of the Moran’s I tests indicate that there is a significantly positive spatial autocorrelation in the isotope ratios (Table 2).

**Table 1:** Linear regression results for δ^2^H, δ^18^O and d-excess May and November 2017 campaign predictions.

Isotope ratio	Campaign	Explanatory variables	df	RSS	R^2^	AIC	F	p-value
δ^2^H	May	MAP + MAT + elev + apan + tocoast + MAH + elev*tocoast + MAP*apan	257	9.38	0.332	1,220.9	16.3	< 0.001
		**elev + tocoast + MAH**	**262**	**9.33**	**0.330**	**1,215.1**	**32.8**	**< 0.001**
	November	MAP + MAT + elev + apan + tocoast + MAH + elev*tocoast + MAP*apan	193	12.80	0.185	1,057.1	5.6	< 0.001
		**elev + tocoast + MAH**	**198**	**12.71**	**0.176**	**1,051.4**	**14.3**	**< 0.001**
δ^18^O	May	MAP + MAT + elev + apan + tocoast + MAO + elev*tocoast + MAP*apan	257	1.58	0.206	254.6	8.5	< 0.001
		**MAP + elev + tocoast + MAO**	**261**	**1.57**	**0.203**	**249.0**	**16.9**	**< 0.001**
	November	MAP + MAT + elev + apan + tocoast + MAO + elev*tocoast + MAP*apan	193	2.36	0.097	361.3	2.6	0.009
		**MAP + elev + tocoast + MAO**	**197**	**2.34**	**0.094**	**355.8**	**5.2**	**< 0.001**
d-Excess	May	MAP + MAT + elev + apan + tocoast + MAD + elev*tocoast + MAP*apan	257	4.40	0.259	809.7	11.4	< 0.001
		**MAP + elev + apan + tocoast + MAD**	**260**	**4.40**	**0.250**	**808.0**	**17.6**	**< 0.001**
	November	MAP + MAT + elev + apan + tocoast + MAD + elev*tocoast + MAP*apan	193	6.55	0.293	781.0	10.2	< 0.001
		**MAP + MAT + apan + tocoast + MAD**	**196**	**6.51**	**0.291**	**777.5**	**16.4**	**< 0.001**

The bold rows indicate the variables that were selected for use in the final universal kriging models.

**Table 2:** Moran’s I test results for δ^2^H, δ^18^O and d-excess May and November 2017 campaign observations.

Isotope ratio	Campaign	Moran’s I coefficient	Test statistic	p-value
δ^2^H	May	2.06	0.344	< 0.001
	November	1.95	0.369	< 0.001
δ^18^O	May	1.43	0.243	< 0.001
	November	1.50	0.300	< 0.001
d-Excess	May	1.29	0.279	< 0.001
	November	1.51	0.314	< 0.001

### Quantifying seasonal variation

**Figure 5:**
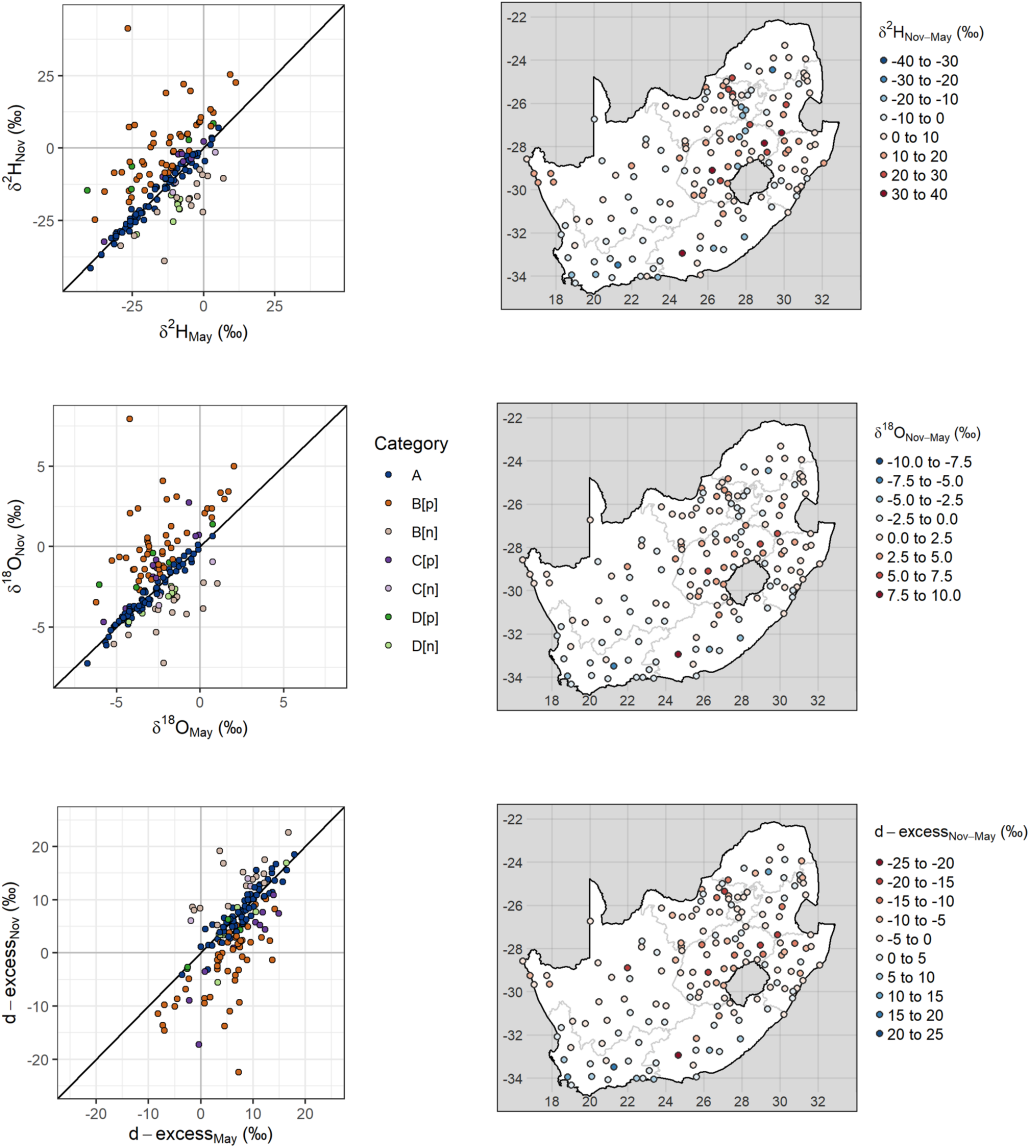
Difference between November and May 2017 δ^2^H (top), δ^18^O (middle) and d-excess (bottom) values in tap water. The categorised seasonal values are plotted against each other with a 1:1 reference line (left) and the difference between the two sampling campaigns shown spatially (right).

**Figure 6:**
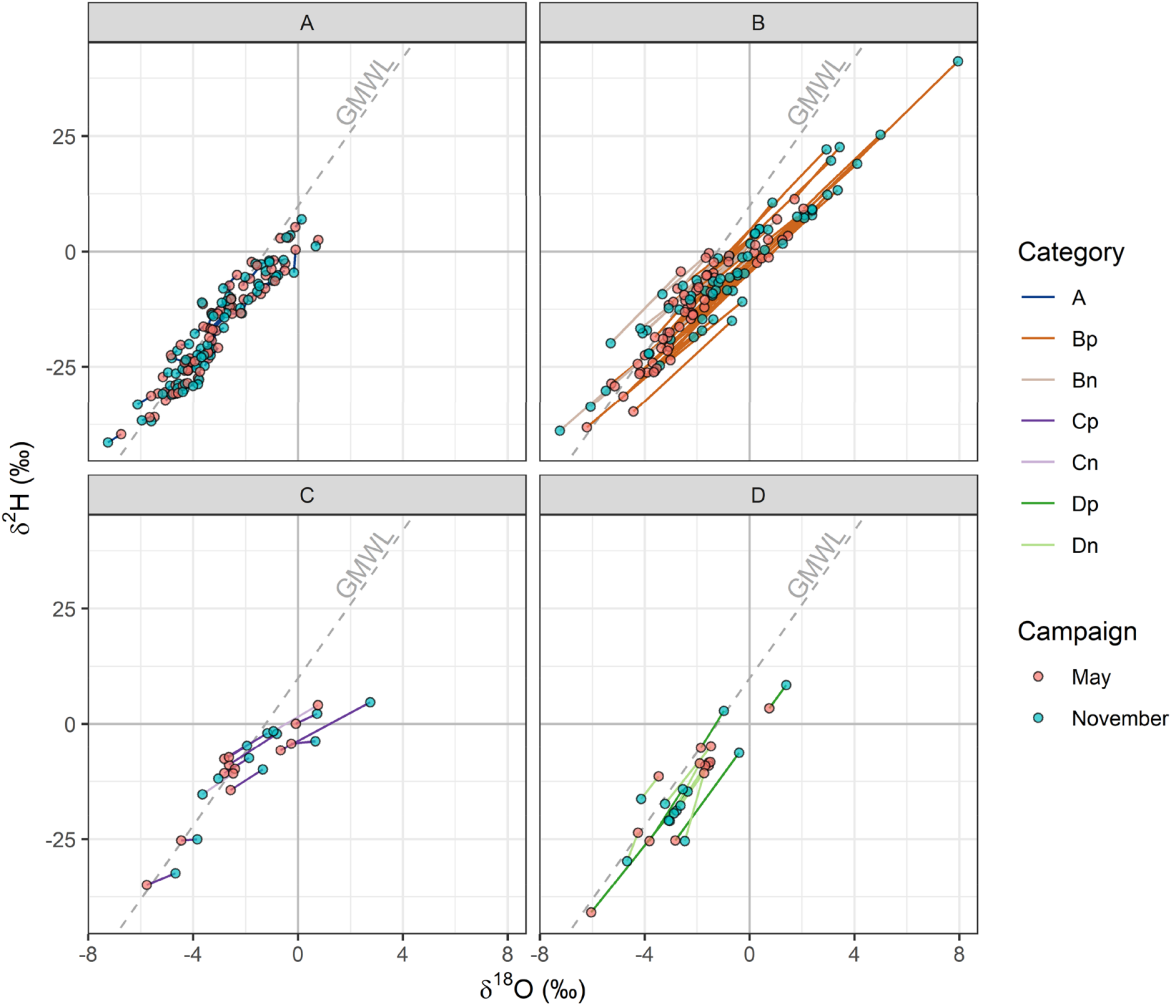
Tap water δ^2^H and δ^18^O values for May (red) and November (blue) 2017 locations that provided samples for both campaigns. The sample pairs are separated according to the nature of the variation between the two campaigns. Category A samples are seasonally invariant; B varied in a manner consistent with evaporation; C had an atypically shallow gradient of variation; and D had an atypically steep gradient of variation. The GMWL is shown by the dashed line for reference.

Seasonally invariant (Category A) samples made up 43% of all repeated sample locations, with categories B, C and D making up 40%, 8% and 9% respectively (Fig. 5). The distributions of category A isotope ratios were consistent with the expectation for meteoric water (Fig. 6). Category B May samples were similar to those of category A, but reflected a greater degree of evapoconcentration and precipitation of recycled continental moisture in November than in May for the positive and negative subcategories, respectively (Fig. 6). Precipitation of recycled continental moisture was evidenced primarily by the more positive d-excess values, some of which were greater than 15‰ (Fig. 5). Evidence of recycled continental moisture precipitation was not observed in either category C or D and considerably negative d-excess values were only observed in some November C_p_ samples (Fig. 6). Despite notable differences between May and November distributions for both D_p_ and D_n_ δ^2^H and δ^18^O values, the distributions of their d-excess values were similar between the two sampling campaigns (Fig. 6). The variation in category D isotope ratios was not consistent with differences between the seasons in the degree of evapoconcentration or proportion of precipitation from recycled continental moisture.

**Figure 7:**
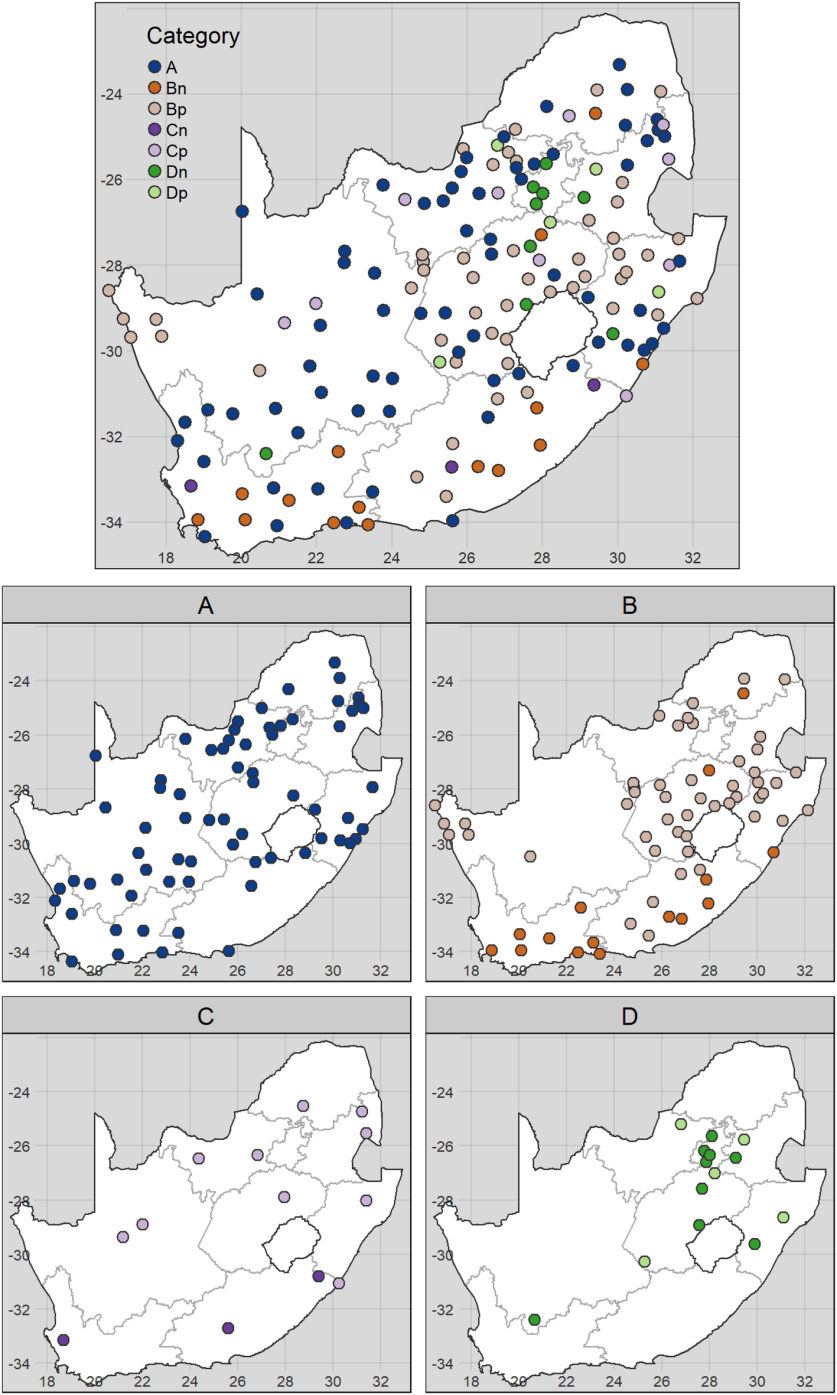
Spatial distribution of the four categories of seasonal isotope variation (**A**–**D**), further distinguished by the direction of seasonal change in the isotope ratios. Categories are defined in as invariant (**A**), variation consistent with evaporation (**B**), and variation inconsistent with evaporation as a result of shallow (**C**) and steep (**D**) gradients of change. Variation is considered positive (p) where isotope ratios are greater in November than in May and vice versa for negative (n) variation.

The distribution of the four categories of seasonal isotope variation varied spatially across the region (Fig. 7). Sample locations in the Northern Cape and North West provinces were dominated by category A variation, sample locations in the Western Cape, Eastern Cape, Free State and KwaZulu-Natal provinces were dominated by category B variation, Gauteng province consisted almost entirely of category D variation and the sample locations in the Limpopo and Mpumalanga had an approximately equal mix of all four categories of variation (see Fig. S1 for cadastral information).

### Interpolation of variation categories

**Figure 8:**
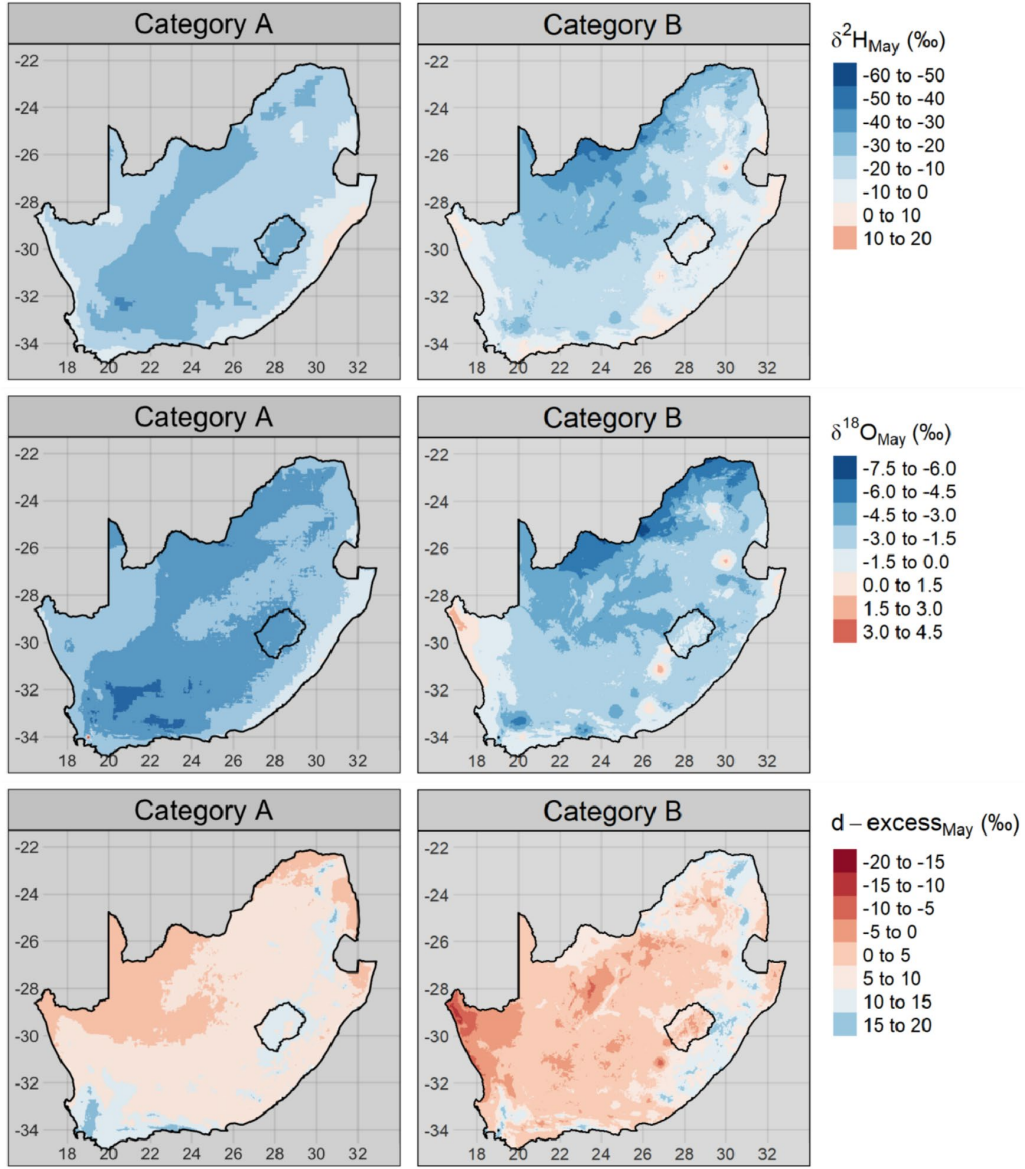
Universal kriging model predictions of δ^2^H (top), δ^18^O (middle) and d-excess (bottom) from the May 2017 category A (right) and B (left) samples. Hatching indicates areas where the model uncertainty is greater than one standard deviation of the underlying data.

**Figure 9:**
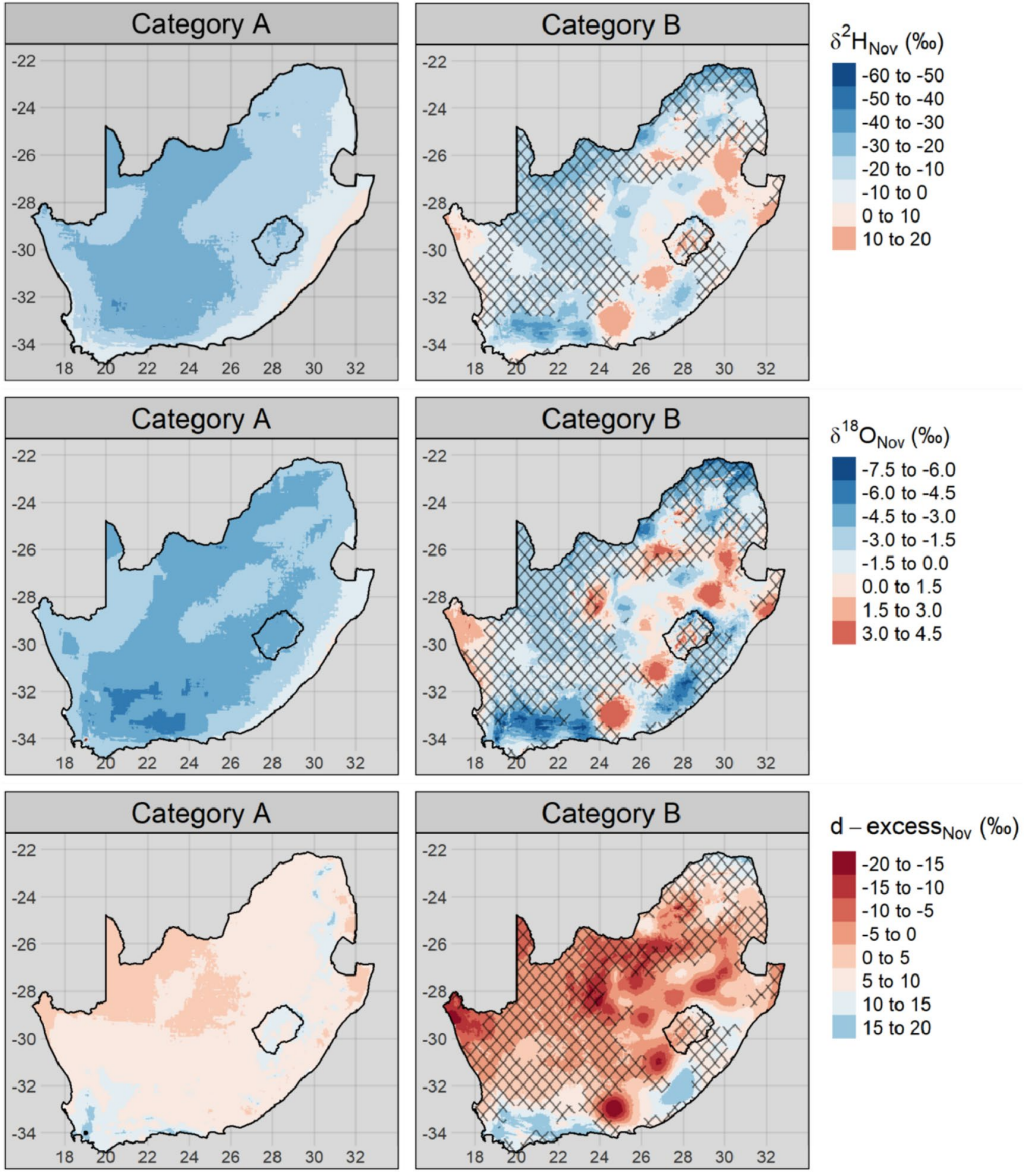
Universal kriging model predictions of δ^2^H (top), δ^18^O (middle) and d-excess (bottom) from the November 2017 category A (right) and B (left) samples. Hatching indicates areas where the model uncertainty is greater than one standard deviation of the underlying data.

Isoscapes for the May (Fig. 8) and November (Fig. 9) δ^2^H, δ^18^O and d-excess values for category A and B sample locations were produced by universal kriging. The density and distribution of category C and D sample locations are too limited to allow for representative kriging of national scale isoscapes.

The spatial variability of category A samples broadly reflected that of the predicted precipitation isotopes (Fig. S3). The δ^2^H and δ^18^O values for category A were more negative in the interior than on the coast; the eastern coast in KwaZulu-Natal province, in particular, had more positive δ^2^H values than the rest of the study area.

Several category B sample locations were discrete outliers, demonstrating various degrees of evapoconcentration. This was evident by ‘bulls-eye’ patterns of atypically positive δ^2^H and δ^18^O values and atypically negative d-excess values (Figs. 8 and 9). These discrete evapoconcentrated outliers were more obvious in the November than the May sampling campaign.

The differences in the spatial coherence of samples from the two categories were evident in the residual error of the universal kriging models (Figs. S7 and S8). The coherence of category A samples were reflected in the low residual error, even in regions with a low density of observations such as the Eastern Cape province (see Fig. 7 for the distribution of category A sample locations). Conversely, the lack of coherence of category B samples was reflected by the high residual error at short distances away from the observed sampling locations. Model uncertainty greater than one standard deviation of the underlying data was especially evident in the November category B isoscapes (Fig. 8). This indicates that there is less confidence in the interpolation of observed isotope ratios beyond the sampling location for this category.

## Discussion

We demonstrate that remote, citizen-sampled seasonal variability of tap water isotopes is potentially an efficient and cost-effective method of identifying and quantitatively characterizing groundwater and surface water dependent tap water sources—the two tap water “worlds”. Seasonal variability in stable water isotope ratios, as defined by the magnitude, gradient and direction of change between May and November, provides a powerful diagnostic tool to help identify the primary source of tap water supply. Sampling locations that exhibited a low magnitude of seasonal variability (Category A; |Δδ^2^H| < 5‰ and |Δδ^18^O| < 0.6‰) are consistent with the expectations for groundwater^8^. Sampling locations that exhibited seasonal variability that approximate local evaporation (Category B; 4 < $\frac{{\Delta \delta^{2} H}}{{\Delta \delta^{18} O}}$ < 7) are consistent with the expectations for surface water^36^.

After disaggregating between surface and groundwater reliant municipalities, considerable distinctions are noted in the spatial variability of δ^2^H and δ^18^O (Figs. 8 and 9). This has direct implications on the application and utility of stable isotopes in tap water for water resource management. Surface-coupled water sources are exposed to risks that are distinct from that of groundwater reliant sources; for example, they are more impacted by silting up due to upstream erosion processes. They are also at greater risk of increased evaporative losses due to climate change. Groundwater sources, by contrast, are more at risk of saltwater intrusion and from dissolving salt from the substrate. Furthermore, this has implications for how stable isotopes are applied in fields such as human (ground and surface water use) and wildlife (surface only) forensics, which rely on the predictability of spatiotemporal variation in stable isotope ratios^37^.

The climatological and topographical explanatory variables used in the kriging models accounted for less variation in the isotope ratios from the November campaign than those of the May campaign (Table 1). This may be a result of the greater degree of evapoconcentration that was observed, on average, in November than in May samples (Fig. 3). The degree of evapoconcentration is dependent, in part, on the surface area to volume ratio of the evaporating water body with greater ratios resulting in a greater proportion of evaporate to residual water than reservoirs with smaller ratios. The spatial distribution of evapoconcentration is therefore not primarily dependent on climatological or topographical variables that vary continuously, but on discrete reservoir-specific characteristics. Therefore, the more that the spatial variation in δ^2^H and δ^18^O values are determined by evapoconcentration, the less the predictive power of climatological and topographical variables or spatial autocorrelation will be. This is also reflected in the greater standard deviations of the category B kriging models for November compared to May in areas with a lower sampling resolution (Figs. S7 and S8). The extrapolation of δ^2^H and δ^18^O values beyond the location of sampling is unreliable for this category (Fig. 8) as the mechanism of variation is dependent on discrete factors such as the properties of the reservoir source. Linear interpolation based on environmental factors and spatial autocorrelation is therefore inappropriate.

The sample locations that demonstrated medium to high seasonal variability (Δδ^2^H| > 5‰ and |Δδ^18^O| > 0.6‰) have several possible interpretations. The source of tap water supply could entirely or partly include surface water reservoirs that experience a greater degree of evapoconcentration in the dry than the wet season, or the source could entirely or partly include non-local water that has stable isotope ratios distinct from that of local water. Non-local water could be distant in space (i.e., inter-basin water transfer from a different catchment area or desalinated ocean water) or distant in time (i.e., fossil groundwater that was recharged under different climatic conditions). The gradient of change in water stable isotopes $\left( {\frac{{\Delta \delta^{2} H}}{{\Delta \delta^{18} O}}} \right)$ can help distinguish between the possible scenarios.

The seasonal variation from local evapoconcentration can only result in isotope ratio changes along the local evaporation line (LEL) in the positive direction^36^. If a given municipality blends some ratio of local ground and surface water, the gradient of change in the isotope ratios will still not deviate from the LEL if all local water sources are recharged by local precipitation. Constrained groundwater aquifers that were recharged under different conditions may have isotope ratios that differ from the local mean, in which case blending or source-switching between the local and non-local water will result in deviations from the LEL. The same is true for inter-basin water transfer (IBWT) schemes^12^.

**Figure 10:**
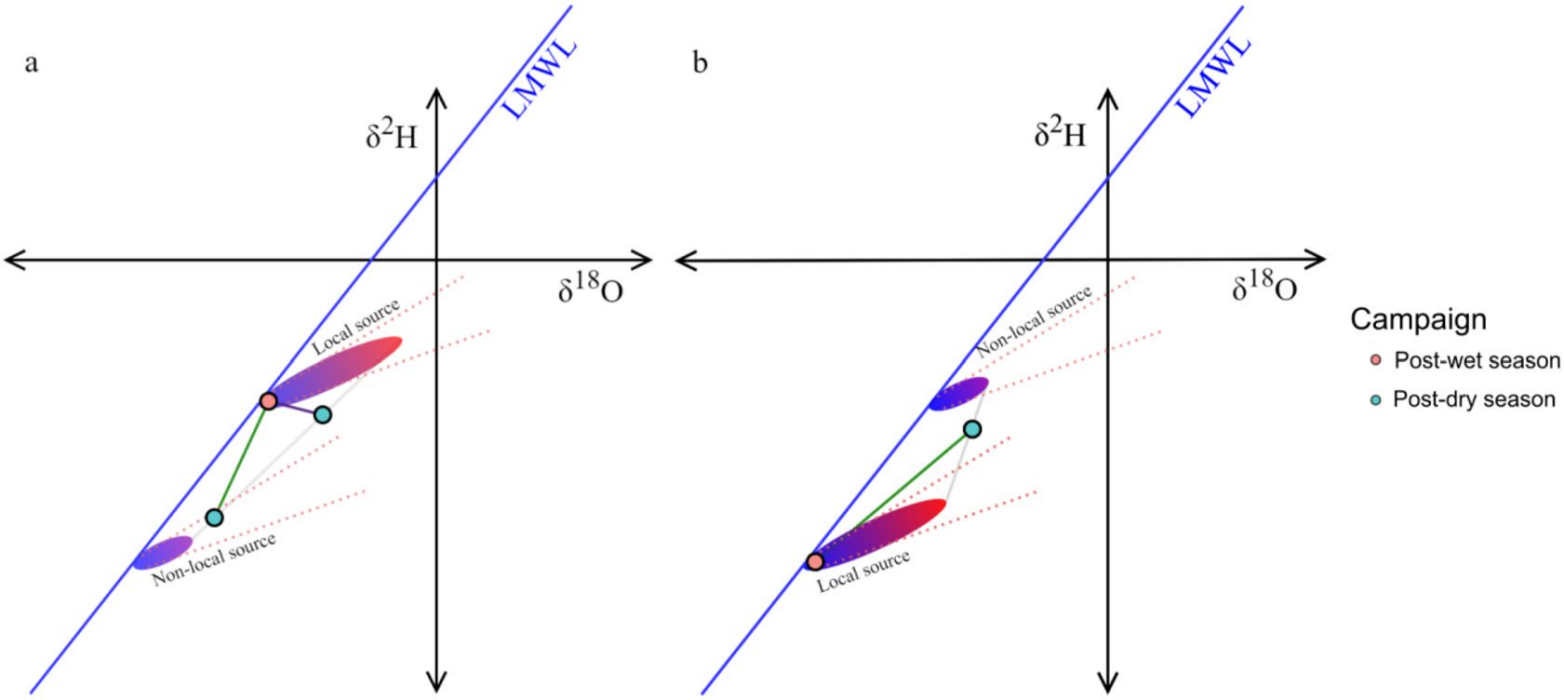
Hypothetical seasonal variation in tap water δ^2^H and δ^18^O resulting from non-local source augmentation in the dry season. Additions from sources that are recharged by more depleted (**a**) and more enriched (**b**) in heavier isotopes in precipitation are presented with the hypothetical post-wet season isotope ratios in orange and two hypothetical post-dry season isotope ratios in blue.

Figure 10 presents some hypothetical seasonal variation scenarios following the blending of isotopically distinct local and non-local water. The assumption is that there is a greater degree of evapoconcentration in the local water reservoirs that require augmentation given that they would be more depleted. If the non-local water source is a constrained groundwater aquifer, rather than from IBWT, then similar patterns in the seasonal variation would occur—the only difference being that the non-local source would experience no evapoconcentration and would be isotopically invariant. The direction of change depends on whether winter or summer is the dry season for the given sample location. Sites in summer rainfall regions would be expected to vary in the opposite direction to those in winter rainfall regions. Sampling locations in the Gauteng province offer an empirical example of an IBWT scheme^4^.

**Figure 11:**
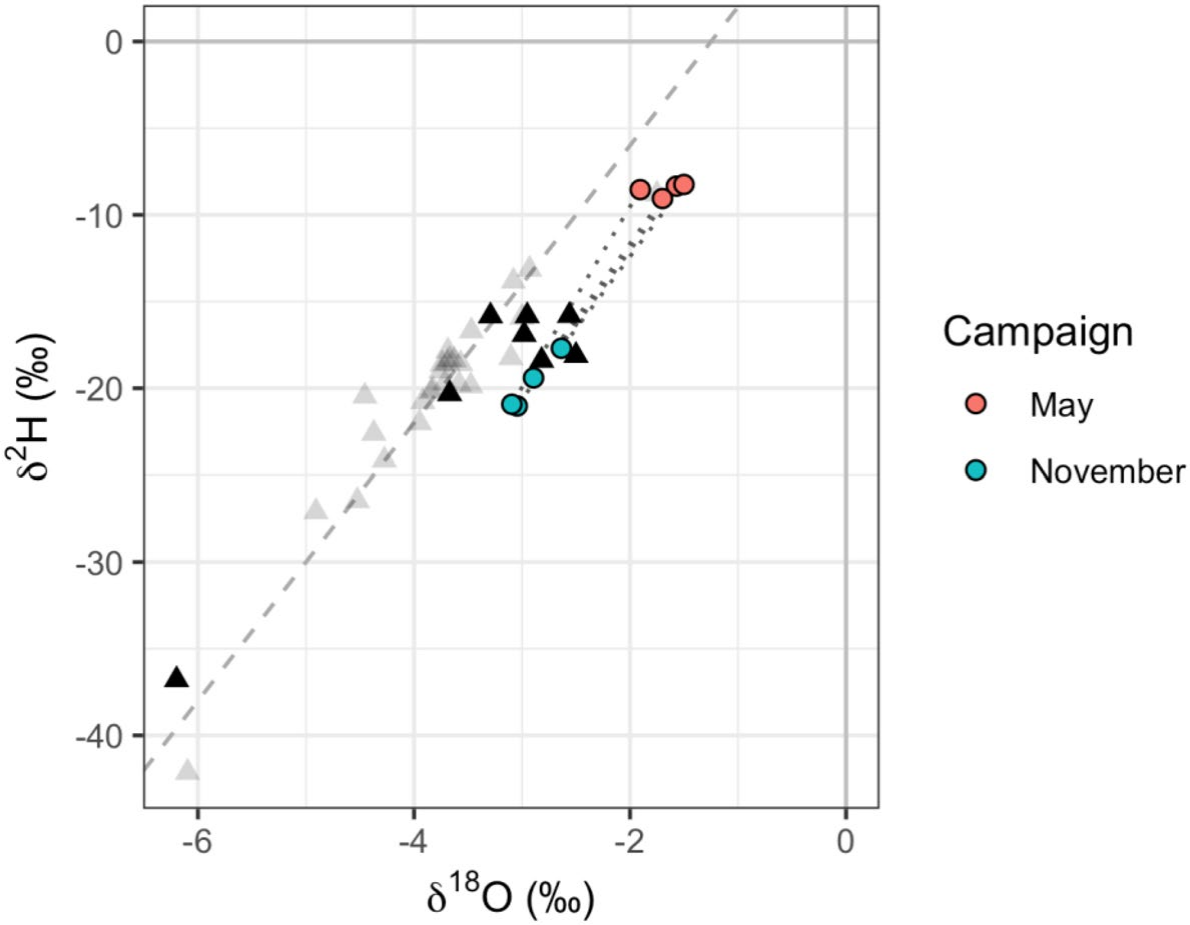
Gauteng δ^2^H and δ^18^O values for the May (red) and November (blue) 2017 sampling campaigns (circles) compared to that of Lesotho (triangles), near Katse Dam (black). The grey triangles show the values for other sample locations in Lesotho not close to Katse Dam (see Fig. S4).

The water requirements of the urban municipalities and industrial sectors in Gauteng are met by a large IBWT scheme sourced from Katse Dam in Lesotho^4^. Two expectations are provided, based on the principles outlined above. The climatic and topographical dynamics between the Gauteng and Lesotho catchments suggest that the scenarios illustrated in Fig. 10a best reflect that of this case study. The degree to which the Gauteng municipalities are dependent on water supply from Lesotho in the dry season suggests that the scenario illustrated with the green line (positive gradient) better reflects this case study than that of the purple line (negative gradient) in Fig. 10a. These two expectations are supported by the direction and the gradient of variation, respectively, in tap water δ^2^H and δ^18^O values between May and November (Fig. 11).

Future studies can improve the interpretation of seasonally variable tap water isotopes by quantifying the spatial and seasonal variability in the LEL gradient. Additionally, further comparisons between the national-scale groundwater isotope ratio predictions based on tap water samples and catchment-scale groundwater isotope data would be valuable to determine the accuracy at scales relevant to local water resource management. Comparisons between the predicted groundwater isotope ratios and that of local modern precipitation would also contribute to determining whether or not the groundwater being extracted was likely recharged recently or is non-renewable^12^. The use of other geochemical tracers may further distinguish between modern and fossil groundwater aquifers^38,39^.

The seasonal variability of δ^2^H, δ^18^O and d-excess values in tap water aid in to their application to urban hydrology. In particular, it has allowed for the identification of the two tap water “worlds”—municipalities that likely rely on groundwater and surface water dependent tap water sources. This has several implications for fields such as water resource management, ecology and forensics^1^. This study has demonstrated that stable H and O isotopes in tap water have the potential to complement traditional water resource management practices. The degree of observed temporal variability suggests that future studies of stable isotopes in tap water need, at a minimum, to incorporate seasonal sampling in order to understand the variable source and transport dynamics of a given location.

## Supplementary information

Supplementary Information.

Supplementary Tables.
