# Supplementary material for: Seasonal variation in tap water δ2H and δ18O isotopes reveals two tap water worlds
Source: Sci Rep. 2020 Aug 11;10:13544. doi: 10.1038/s41598-020-70317-2 (PMC7421565; doi:10.1038/s41598-020-70317-2)
Supplement: Supplementary file 1 — Supplementary Information. [file 41598_2020_70317_MOESM1_ESM.docx]

**Supplementary Information**

**Title**: Seasonal variation in tap water $\delta$^2^H and $\delta$^18^O isotopes reveals two tap water worlds.

**Authors**: Ruan F. de Wet, Adam G. West and Chris Harris

### *Corresponding author*

Correspondence to Ruan F. de Wet. Email: rfdewet@gmail.com

**Seasonal variation of isotopes in tap water:**

Seasonal variability of isotopes in tap water is determined by that of the source of municipal supply, and whether or not municipal reliance on each source varies between seasons. Reliance on groundwater supply, for example, can be distinguished from that of continental surface water because groundwater aquifers are seasonally invariant with respect to their δ^2^H and δ^18^O values^1,2^. Some surface water reservoirs, however, may be seasonally invariant, depending on the proportion of water lost to evaporation.

The proportion, not the amount, of water lost to evaporation determines the degree of evapoconcentration. Ocean waters, for example, undergo a high degree of evaporation, in absolute terms, but relative to the total volume of the ocean, the proportion of water lost to evaporation is negligible. Terrestrial surface water reservoirs, however, can lose a significant proportion of their total volume to evaporation, which results in increases of the δ^2^H and δ^18^O values of the residual water. In regions with a high degree of seasonality of precipitation amount, the proportion of the total surface water volume that is evaporated will be greatest at the end of the dry season. The proportion of evaporation also relies on the surface area to volume ratio of the reservoir, with a greater ratio resulting in more evapoconcentration, and the potential evaporation rate, which in turn is dependent on temperature, relative humidity and wind speed. The greater the evapoconcentration, the greater the enrichment of heavier isotopes in the corresponding tap water (more positive δ^2^H and δ^18^O values) and the more negative the deuterium-excess value (Figure 1).

Seasonal variation of stable isotopes in tap water need not be related to evapoconcentration, however. Some municipalities may rely on different sources in the dry than in the wet season. Non-local water use, from inter-basin water transfers or fossil groundwater mining, can result in seasonally variable isotopes in tap water, depending on how the δ^2^H and δ^18^O values differ between the two sources. Isotopic variation due to evaporation can be distinguished from that of source switching because evapoconcentration results in variation along the gradient of the LEL in a predictable direction. If the gradient and direction of seasonal isotope variation are atypical of evapoconcentration, then it would most likely have resulted from source processes. As yet, most of these inferences remain untested. Some of them are tested for the first time in this study.

**Supplementary References:**

1. Gat, J. R. Comments on the stable isotope method in regional groundwater investigations. *Water Resour. Res.* **7**, (1971).

2. Landwehr, J. M., Coplen, T. B. & Stewart, D. W. Spatial, seasonal, and source variability in the stable oxygen and hydrogen isotopic composition of tap waters throughout the USA. *Hydrol. Process.* **28**, 5382–5422 (2014).

3. Schulze, R. E. *South African atlas of climatology and agrohydrology*. *WRC Report 1489/1/06* (2007).

# **Appendix A: Study area**

| 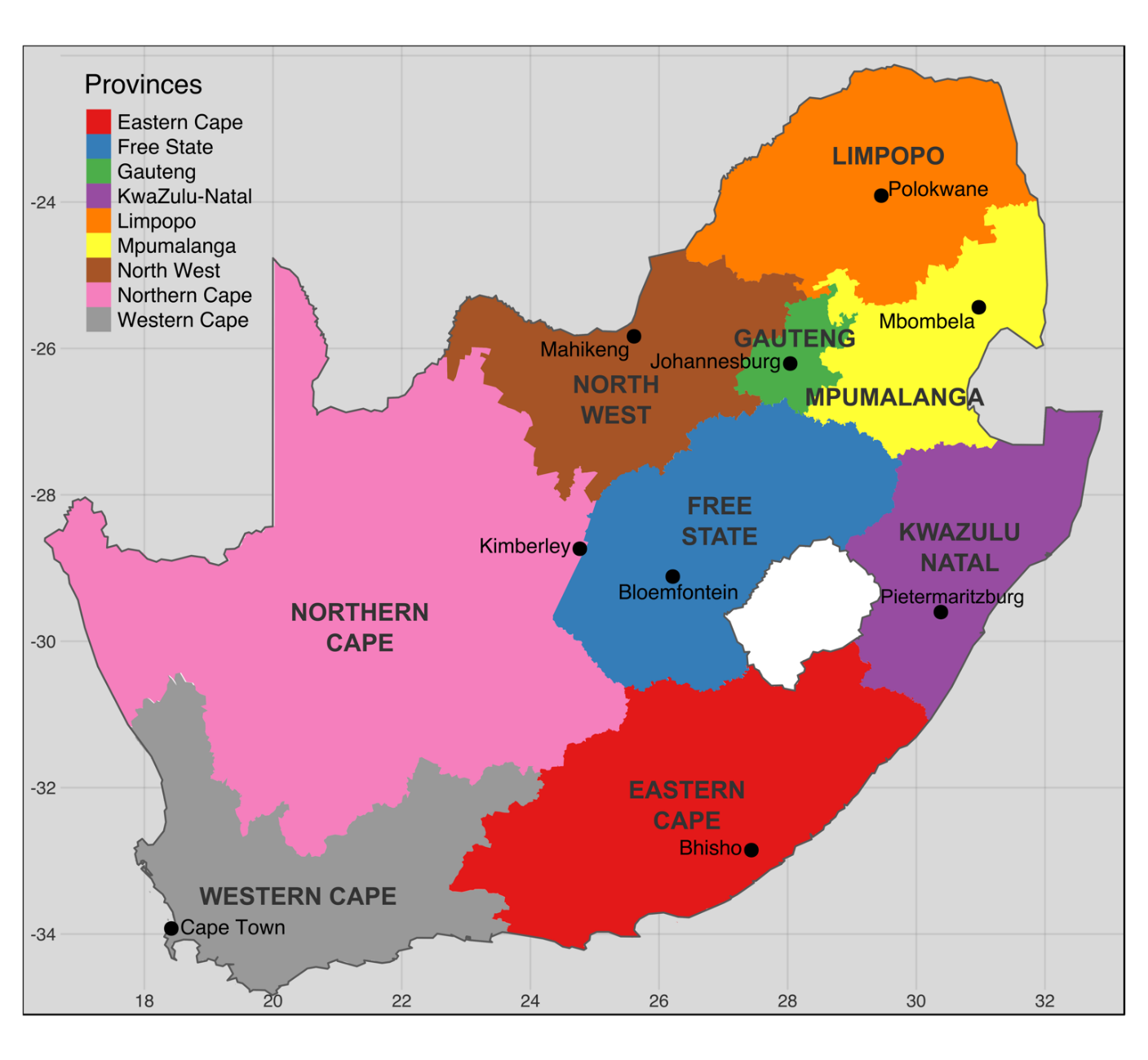 |
| --- |
| **Figure S1.** Provinces and their capital cities in the Republic of South Africa. |


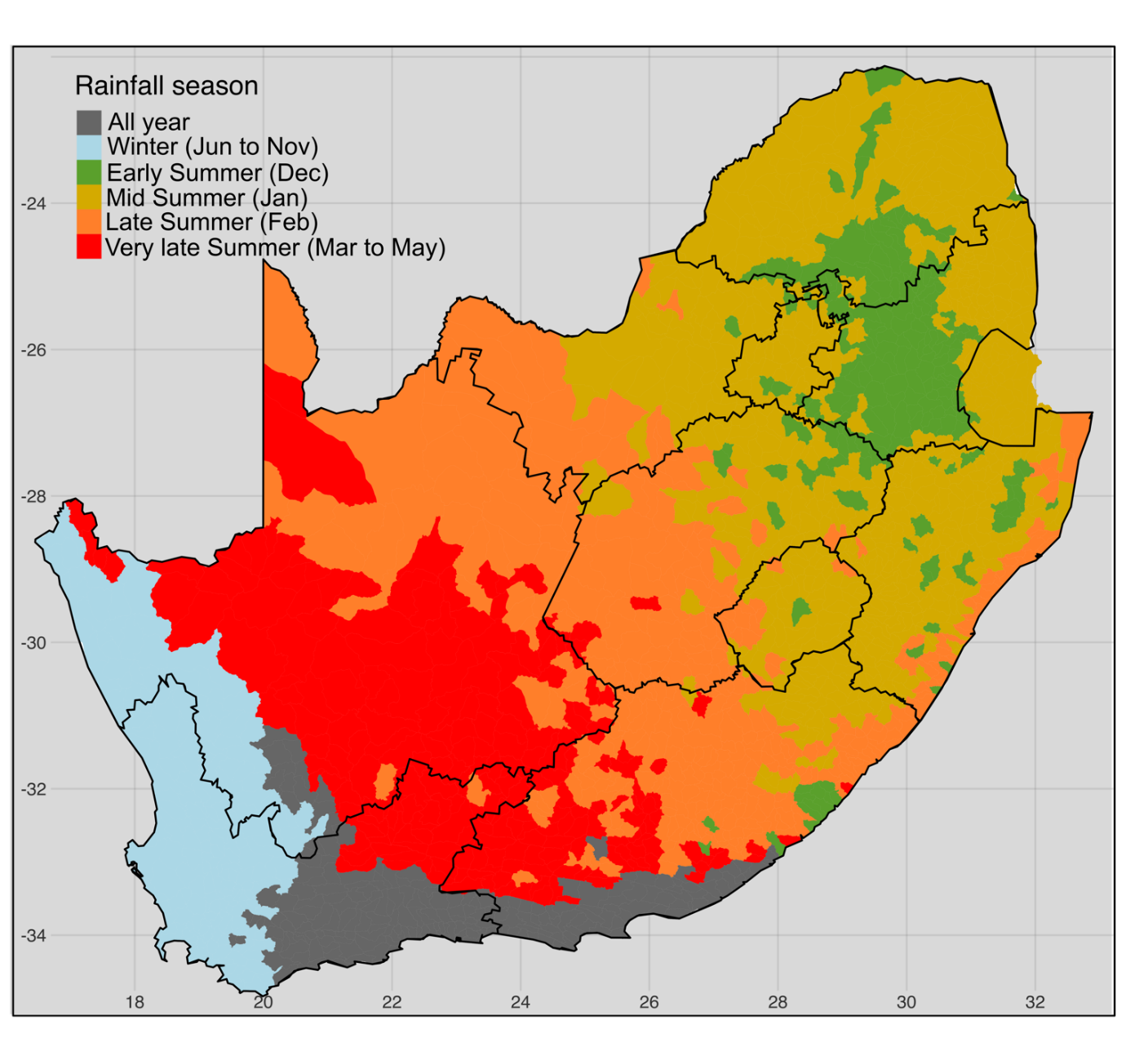


**Figure S2.** Rainfall seasons (from Schulze, 2007) separated into all year (grey), winter (blue), early summer (green), middle summer (yellow), late summer (orange) and very late summer (red).

| 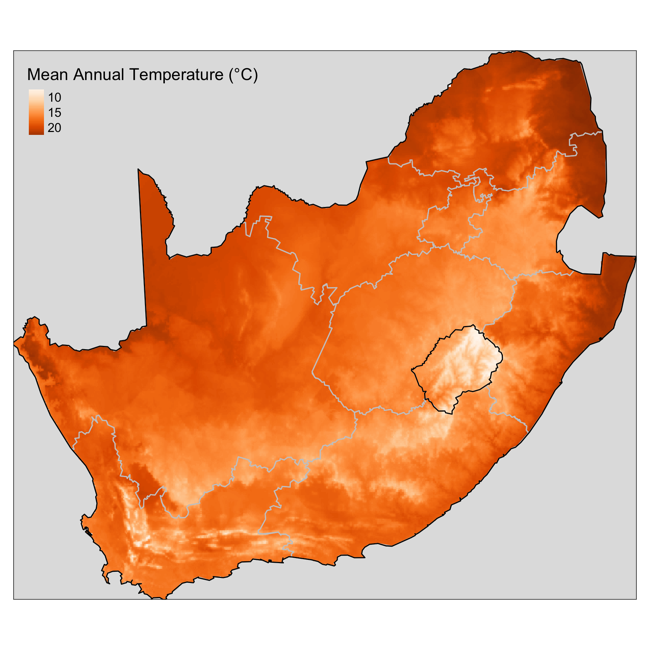 | 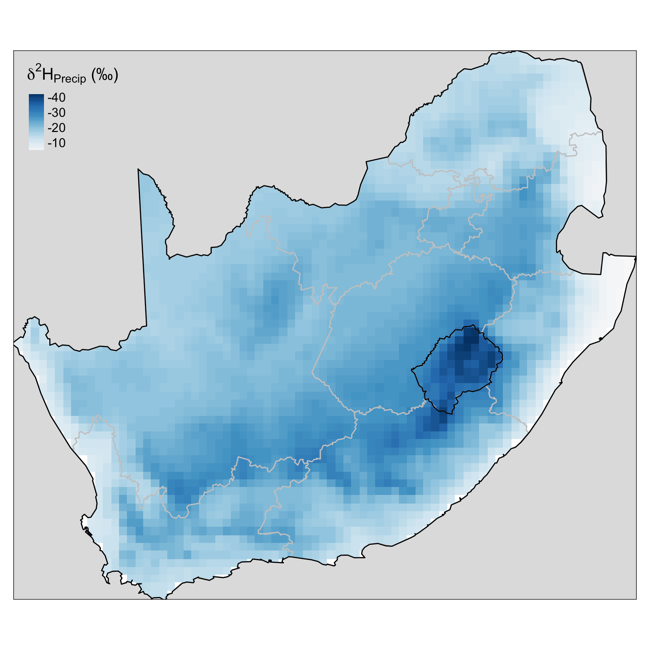 |
| --- | --- |
| 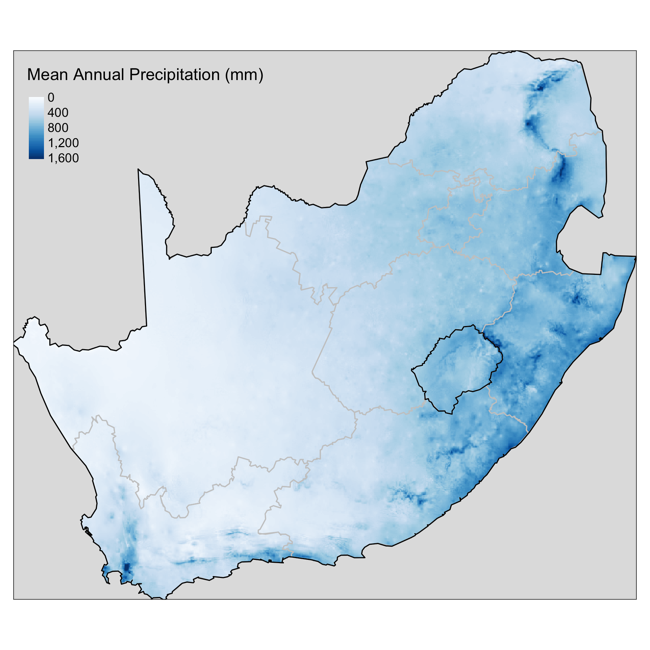 | 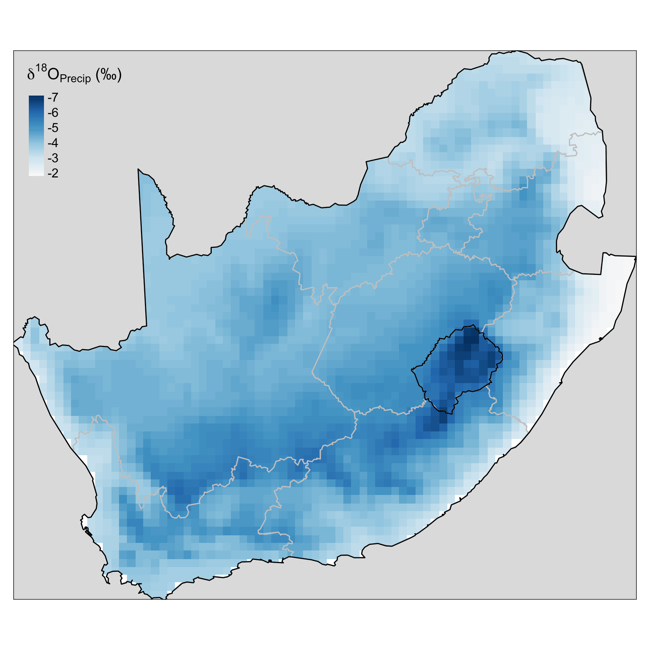 |
| 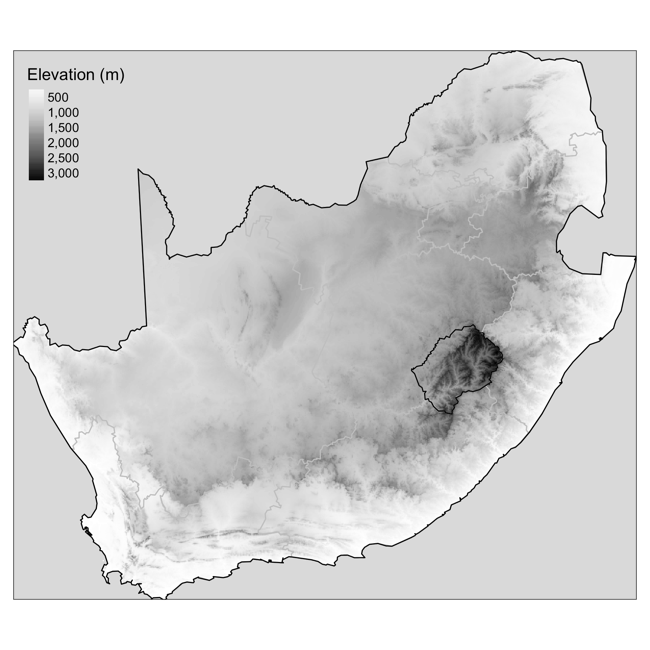 | 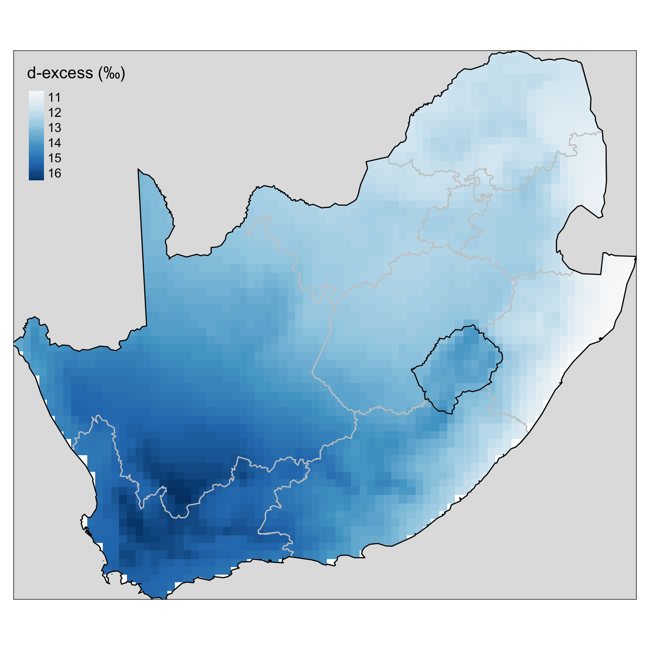 |
| 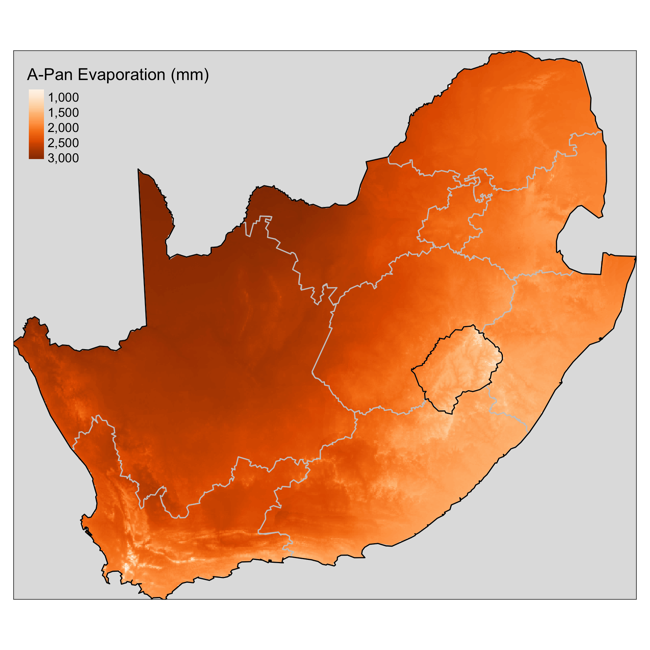 | 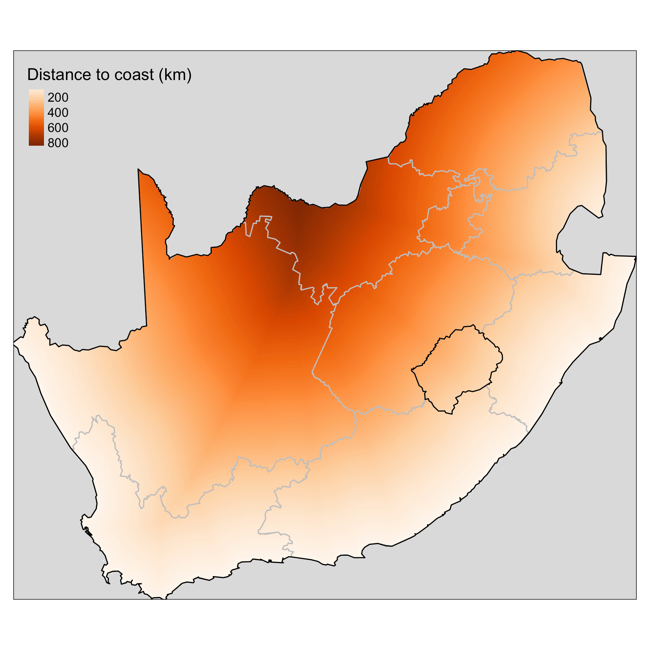 |
| **Figure S3.** Spatial distribution of the explanatory variables applied to the universal kriging models. See methods section for the data sources. | |

# **Appendix B: Sample kit enclosed letter**


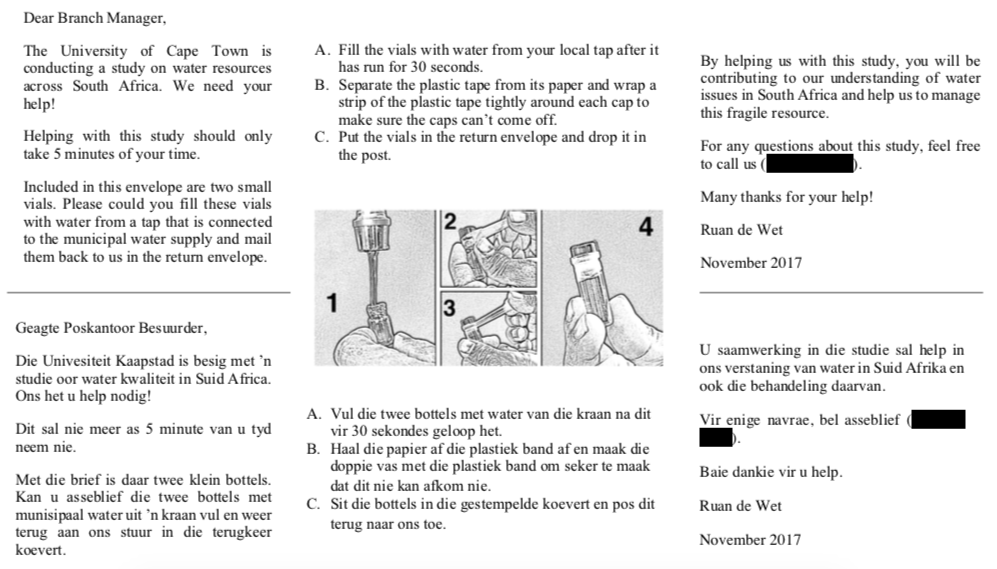


**Figure S4.** Letter describing the tap water sampling method enclosed in the return-mail sampling kits.

# **Appendix C: Krige variograms**

| 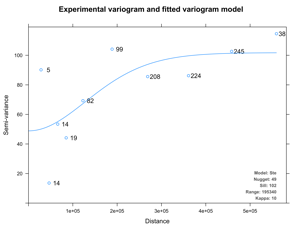 | 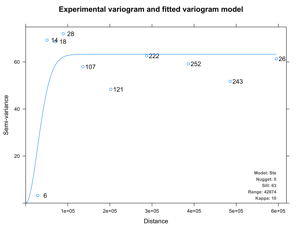 |
| --- | --- |
| 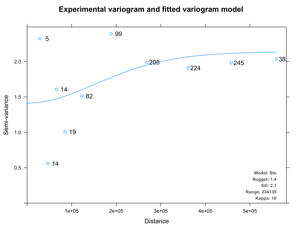 | 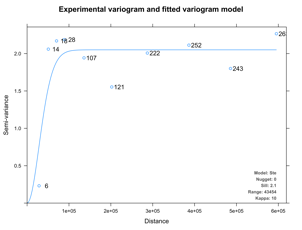 |
| 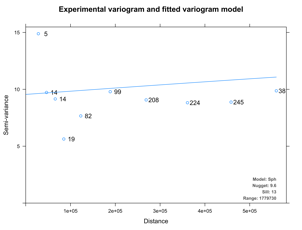 | 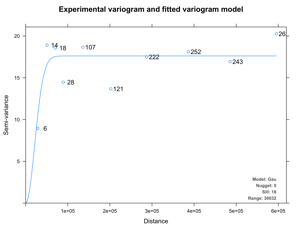 |

**Figure S5**. May category A (left) and category B (right) 2017 sampling campaign universal kriging experimental and fitted variogram models for δ^2^H (top), δ^18^O (middle) and d-excess (bottom).

| 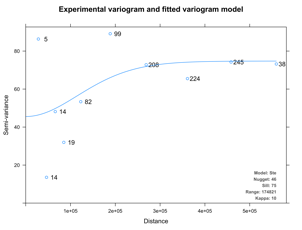 | 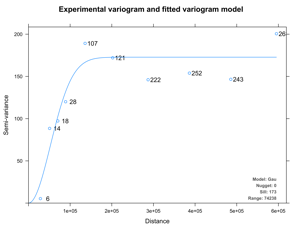 |
| --- | --- |
| 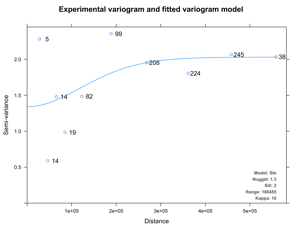 | 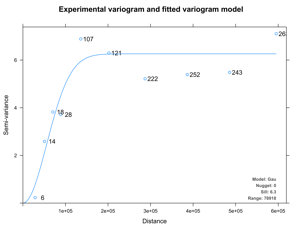 |
| 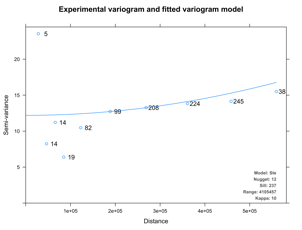 | 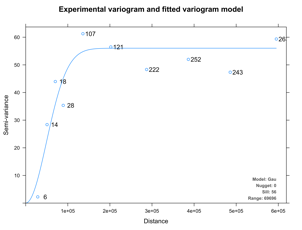 |

**Figure S6**. November category A (left) and category B (right) 2017 sampling campaign universal kriging experimental and fitted variogram models for δ^2^H (top), δ^18^O (middle) and d-excess (bottom).

# **Appendix D: Krige model standard deviations**

| 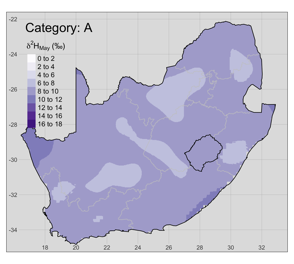 | 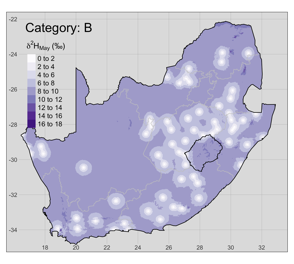 |
| --- | --- |
| 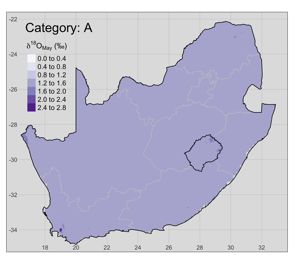 | 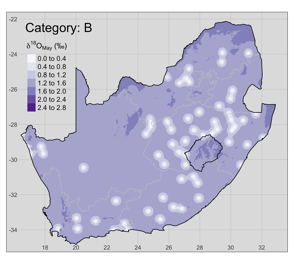 |
| 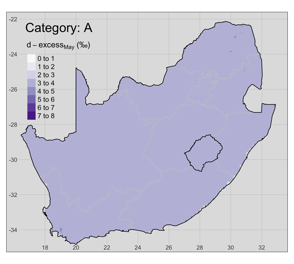 | 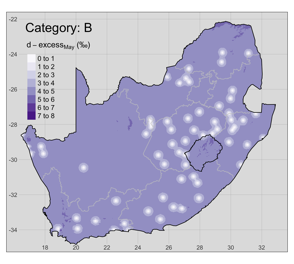 |

**Figure S8**. Universal kriging model standard deviations for δ^2^H (top), δ^18^O (middle) and d-excess (bottom) from the May 2017 category A (right) and B (left) samples.

| 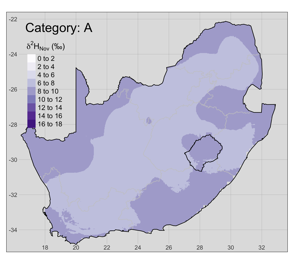 | 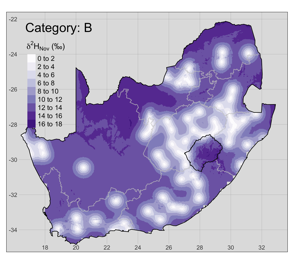 |
| --- | --- |
| 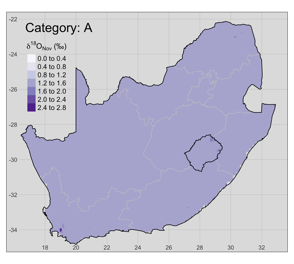 | 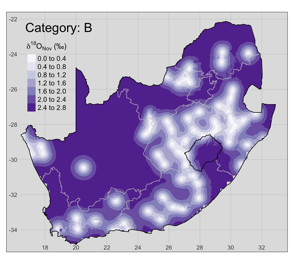 |
| 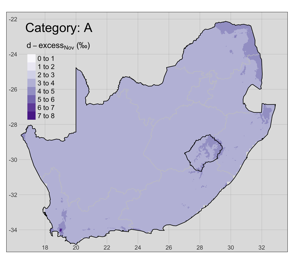 | 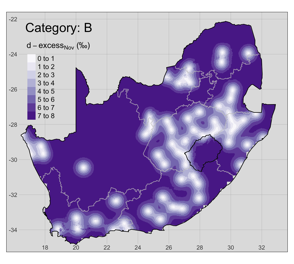 |

**Figure S9**. Universal kriging model standard deviations for δ^2^H (top), δ^18^O (middle) and d-excess (bottom) from the November 2017 category A (right) and B (left) samples.
