# Supplementary material for: Seasonal variation in tap water δ2H and δ18O isotopes reveals two tap water worlds
Source: Sci Rep. 2020 Aug 11;10:13544. doi: 10.1038/s41598-020-70317-2 (PMC7421565; doi:10.1038/s41598-020-70317-2)
Supplement: Supplementary file 2 — Supplementary Tables. [file 41598_2020_70317_MOESM2_ESM.docx]

**Supplementary Data Table**

**Title**: Seasonal variation in tap water $\delta$^2^H and $\delta$^18^O isotopes reveals two tap water worlds.

**Authors**: Ruan F. de Wet, Adam G. West and Chris Harris

### *Corresponding author*

Correspondence to Ruan F. de Wet. Email: rfdewet@gmail.com

**Table S1**. Isotope analysis results (δ^2^H, δ^18^O and d-excess in ‰), South African Post Office (SAPO) code and GPS coordinates of every post office included in the May and November 2017 sampling campaigns.

| Name | Province | SAPO Code | Longitude | Latitude | May 2017 | | | November 2017 | | |
| --- | --- | --- | --- | --- | --- | --- | --- | --- | --- | --- |
|  |  |  |  |  | **δ^18^O** | **δ^2^H** | **d-excess** | **δ^18^O** | **δ^2^H** | **d-excess** |
| ABERDEEN | EASTERN CAPE | 60004 | 24.0622 | -32.4778 | -3.96 | -21.4 | 10.3 | -4.00 | -22.6 | 9.4 |
| ACORNHOEK | MPUMALANGA | 10196 | 31.0500 | -24.5991 | -3.28 | -13.4 | 12.8 | -3.48 | -16.5 | 11.3 |
| ADELAIDE | EASTERN CAPE | 60100 | 26.2966 | -32.7068 | -2.30 | -10.4 | 8.0 | 1.03 | 7.0 | -1.3 |
| ALBERTINIA | WESTERN CAPE | 60301 | 21.5864 | -34.2040 | -3.71 | -18.6 | 11.0 | -4.84 | -21.7 | 17.0 |
| ALEXANDERBAY | NORTHERN CAPE | 60407 | 16.4805 | -28.5958 | 2.96 | 12.3 | -11.4 | 1.45 | 3.4 | -8.2 |
| ALGOAPARK | EASTERN CAPE | 60502 | 25.5621 | -33.9016 | -2.02 | -8.0 | 8.2 |  |  |  |
| ALICE | EASTERN CAPE | 60551 | 26.8382 | -32.7891 | -3.11 | -12.3 | 12.6 | -1.39 | -2.3 | 8.7 |
| ALICEDALE | EASTERN CAPE | 60608 | 26.0829 | -33.3163 | 4.78 | 25.8 | -12.5 | 3.54 | 19.6 | -8.7 |
| ALIWAL NORTH | EASTERN CAPE | 60657 | 26.7108 | -30.6899 | -1.92 | -10.5 | 4.9 | -2.20 | -13.3 | 4.3 |
| AMALINDA | EASTERN CAPE | 60673 | 27.8725 | -32.9956 | -0.02 | 4.7 | 4.9 | 0.36 | 5.3 | 2.4 |
| AMSTERDAM | MPUMALANGA | 10598 | 30.6612 | -26.6232 | -1.28 | -3.8 | 6.4 | -2.46 | -9.8 | 9.9 |
| ATAMELANG | NORTH WEST | 10900 | 25.3692 | -26.5022 | -4.58 | -28.6 | 8.0 | -4.70 | -28.9 | 8.6 |
| AUGRABIES | NORTHERN CAPE | 90123 | 20.4289 | -28.6660 | 0.65 | 1.1 | -4.1 | 0.76 | 2.5 | -3.6 |
| AZAADVILLE | GAUTENG | 10934 | 27.7580 | -26.1702 | -3.04 | -21.0 | 3.3 | -1.57 | -8.3 | 4.2 |
| BA-PHALABORWA | LIMPOPO | 29678 | 31.1378 | -23.9498 | -0.30 | -1.3 | 1.1 | -2.26 | -10.6 | 7.5 |
| BADPLAAS | MPUMALANGA | 10959 | 30.5638 | -25.9572 |  |  |  |  |  |  |
| BAMOKGOKO | MPUMALANGA | 95780 | 28.5354 | -25.1033 |  |  |  | -3.13 | -16.0 | 9.0 |
| BARKLY EAST | EASTERN CAPE | 61055 | 27.5946 | -30.9669 | -1.12 | -5.9 | 3.1 | -2.49 | -13.1 | 6.9 |
| BARKLY WEST | NORTHERN CAPE | 90180 | 24.5199 | -28.5381 | -0.29 | -10.9 | -8.6 | -3.04 | -23.5 | 0.7 |
| BEAUFORT WEST | WESTERN CAPE | 61303 | 22.5826 | -32.3508 | -3.86 | -22.1 | 8.8 | -2.69 | -16.3 | 5.3 |
| BERGVILLE | KWAZULU-NATAL | 40595 | 29.3522 | -28.7311 | -1.29 | -6.4 | 3.9 | -2.76 | -8.2 | 13.9 |
| BETHLEHEM | FREE STATE | 50633 | 28.3096 | -28.2336 | -2.68 | -11.7 | 9.8 | -2.54 | -11.9 | 8.4 |
| BLOEMFONTEIN | FREE STATE | 50842 | 26.2228 | -29.1183 | 2.36 | 7.9 | -11.0 | -3.71 | -24.1 | 5.6 |
| BONNIEVALE | WESTERN CAPE | 62258 | 20.1022 | -33.9395 | -2.25 | -9.6 | 8.4 | 0.19 | 1.4 | -0.1 |
| BOSBOKRAND | MPUMALANGA | 12630 | 31.0694 | -24.8337 | -1.41 | -2.7 | 8.6 | -1.86 | -5.9 | 9.0 |
| BOTHAVILLE | FREE STATE | 51194 | 26.6161 | -27.3933 | -1.53 | -7.0 | 5.3 | -1.43 | -9.2 | 2.3 |
| BOYNE | LIMPOPO | 12793 | 29.7991 | -23.9457 | -2.69 | -11.3 | 10.2 | -3.29 | -14.2 | 12.1 |
| BRANDFORT | FREE STATE | 51338 | 26.4587 | -28.7020 |  |  |  |  |  |  |
| BRANDVLEI | NORTHERN CAPE | 62505 | 20.4875 | -30.4650 | -1.31 | -8.4 | 2.0 | -2.17 | -13.6 | 3.8 |
| BREDASDORP | WESTERN CAPE | 62554 | 20.0387 | -34.5320 |  |  |  | -2.42 | -9.7 | 9.6 |
| BRITS | NORTH WEST | 13158 | 27.7784 | -25.6353 | -1.58 | -8.7 | 4.0 | -1.78 | -9.9 | 4.4 |
| BRITSTOWN | NORTHERN CAPE | 90727 | 23.5028 | -30.5884 | -4.02 | -29.1 | 3.0 | -4.23 | -28.5 | 5.3 |
| BRONKHORSTSPRUIT | GAUTENG | 13359 | 28.7449 | -25.8093 |  |  |  |  |  |  |
| BULTFONTEIN | FREE STATE | 51400 | 26.1538 | -28.2920 | -0.47 | -5.2 | -1.5 | -2.25 | -14.6 | 3.4 |
| BULWER | KWAZULU-NATAL | 40835 | 29.7684 | -29.8065 | -3.06 | -10.8 | 13.6 |  |  |  |
| BURGERSFORT | MPUMALANGA | 13550 | 30.3264 | -24.6685 | -2.01 | -9.9 | 6.1 |  |  |  |
| CALVINIA | NORTHERN CAPE | 63104 | 19.7729 | -31.4749 | -3.89 | -25.3 | 5.8 | -4.22 | -25.8 | 8.0 |
| CARNARVON | NORTHERN CAPE | 63354 | 22.1282 | -30.9697 | -4.43 | -25.4 | 10.0 | -4.11 | -23.9 | 9.0 |
| CAROLINA | MPUMALANGA | 13752 | 30.1166 | -26.0695 | 3.10 | 19.7 | -5.1 | -1.40 | -4.6 | 6.6 |
| CENTRAHIL | EASTERN CAPE | 63602 | 25.6107 | -33.9640 | -1.11 | -2.3 | 6.6 | -1.03 | -3.8 | 4.5 |
| CEZA | KWAZULU-NATAL | 41024 | 31.3756 | -27.9941 | -1.17 | -2.0 | 7.4 | -2.81 | -7.6 | 14.9 |
| CHROMITE | LIMPOPO | 13995 | 27.2852 | -24.8261 | -0.65 | -8.5 | -3.2 | -4.84 | -31.4 | 7.3 |
| CITRUSDAL | WESTERN CAPE | 68392 | 19.0112 | -32.5882 | -4.63 | -21.5 | 15.5 | -4.86 | -22.5 | 16.4 |
| CLANWILLIAM | WESTERN CAPE | 63757 | 18.8900 | -32.1867 |  |  |  | -2.37 | -11.3 | 7.7 |
| CLOCOLAN | FREE STATE | 51547 | 27.5680 | -28.9148 | -2.47 | -25.3 | -5.5 | -1.74 | -10.7 | 3.3 |
| COFIMVABA | KWAZULU-NATAL | 44365 | 29.7813 | -28.5591 | 1.40 | 12.0 | 0.9 |  |  |  |
| COLIGNY | NORTH WEST | 14319 | 26.3184 | -26.3311 | -3.86 | -24.3 | 6.5 | -4.20 | -25.7 | 7.9 |
| CRADOCK | EASTERN CAPE | 64451 | 25.6171 | -32.1660 | -1.42 | -9.1 | 2.3 | -3.32 | -19.0 | 7.6 |
| DA GAMASKOP | WESTERN CAPE | 64637 | 22.1175 | -34.1747 | -1.87 | -3.4 | 11.5 |  |  |  |
| DANIELSKUIL | NORTHERN CAPE | 91029 | 23.5466 | -28.1811 | -5.96 | -36.7 | 11.0 | -5.67 | -35.9 | 9.4 |
| DE AAR | NORTHERN CAPE | 91086 | 24.0131 | -30.6519 | -4.33 | -28.7 | 6.0 | -4.41 | -29.1 | 6.2 |
| DEWETSDORP | FREE STATE | 52104 | 26.6630 | -29.5840 | 0.35 | 4.9 | 2.1 | -3.15 | -17.5 | 7.6 |
| DIE BOORD | WESTERN CAPE | 82440 | 18.8520 | -33.9440 | -4.19 | -16.7 | 16.8 | -0.82 | -2.2 | 4.3 |
| DINOKANA | NORTH WEST | 15578 | 25.8680 | -25.4517 | -4.80 | -28.3 | 10.1 |  |  |  |
| DOUGLAS | NORTHERN CAPE | 91383 | 23.7752 | -29.0541 | -0.97 | -6.3 | 1.4 | -0.51 | -2.6 | 1.5 |
| DUKUZA | KWAZULU-NATAL | 98381 | 29.2088 | -28.7494 | -3.30 | -13.7 | 12.6 | -2.99 | -12.8 | 11.2 |
| DUNDEE | KWAZULU-NATAL | 41765 | 30.2335 | -28.1629 | 0.20 | 4.0 | 2.3 | -1.96 | -7.8 | 7.9 |
| DYSSELSDORP | WESTERN CAPE | 65508 | 22.4380 | -33.5748 |  |  |  | -6.87 | -37.8 | 17.2 |
| ELLIOT | EASTERN CAPE | 65854 | 27.8508 | -31.3335 | -3.93 | -17.1 | 14.4 | -2.51 | -9.4 | 10.6 |
| ERMELO | MPUMALANGA | 16981 | 29.9841 | -26.5220 | 3.43 | 22.6 | -4.9 | 1.70 | 11.4 | -2.3 |
| ESHOWE | KWAZULU-NATAL | 42201 | 31.4675 | -28.8976 | -1.19 | -1.6 | 7.9 |  |  |  |
| ESTCOURT | KWAZULU-NATAL | 42243 | 29.8742 | -29.0055 | -2.04 | -6.2 | 10.1 | -2.93 | -10.9 | 12.5 |
| EXCELSIOR | FREE STATE | 52597 | 27.0618 | -28.9412 | -1.44 | -9.6 | 2.0 | -4.01 | -22.5 | 9.6 |
| FAURESMITH | FREE STATE | 52803 | 25.3154 | -29.7483 | -1.82 | -14.6 | -0.1 | -3.09 | -20.6 | 4.1 |
| FICKSBURG | FREE STATE | 52878 | 27.8769 | -28.8697 | -2.26 | -12.3 | 5.7 |  |  |  |
| FLAGSTAFF | EASTERN CAPE | 66358 | 29.4957 | -31.0801 | -3.31 | -8.4 | 18.1 |  |  |  |
| FOURIESBURG | FREE STATE | 52941 | 28.2080 | -28.6211 | -1.71 | -8.5 | 5.2 | -2.40 | -12.4 | 6.9 |
| FRANKFORT | FREE STATE | 53082 | 28.4925 | -27.2759 |  |  |  | -2.79 | -12.5 | 9.8 |
| FRANSCHHOEK | WESTERN CAPE | 66559 | 19.1205 | -33.9115 | -5.10 | -22.9 | 17.9 |  |  |  |
| FRASERBURG | NORTHERN CAPE | 66606 | 21.5127 | -31.9137 | -3.60 | -22.5 | 6.3 | -3.45 | -21.9 | 5.7 |
| GALLO MANOR | GAUTENG | 18113 | 28.0744 | -26.0667 |  |  |  | -1.74 | -9.5 | 4.5 |
| GLEN HARMONY | FREE STATE | 53363 | 26.8819 | -28.0799 |  |  |  | -1.43 | -8.1 | 3.3 |
| GOMPIES | LIMPOPO | 18493 | 29.4039 | -24.4508 | -3.84 | -22.1 | 8.6 | 0.17 | -0.2 | -1.6 |
| GRAAFF-REINET | EASTERN CAPE | 67205 | 24.5341 | -32.2519 | 9.27 | 54.4 | -19.8 |  |  |  |
| GRAHAMSTOWN | EASTERN CAPE | 67359 | 26.5258 | -33.3111 | 0.05 | 4.0 | 3.6 | 1.91 | 12.4 | -2.9 |
| GRASKOP | MPUMALANGA | 18485 | 30.8402 | -24.9322 | -1.06 | -7.9 | 0.6 |  |  |  |
| GREYTOWN | KWAZULU-NATAL | 42725 | 30.5935 | -29.0602 | -1.12 | -2.0 | 7.0 | -1.58 | -3.0 | 9.6 |
| GRIEKWASTAD | NORTHERN CAPE | 91623 | 23.2513 | -28.8483 | -5.36 | -35.9 | 7.0 |  |  |  |
| GROBLERSHOOP | NORTHERN CAPE | 91680 | 21.9842 | -28.8955 | 2.75 | 4.8 | -17.2 | -0.67 | -5.7 | -0.4 |
| HAMMANSKRAAL | LIMPOPO | 19130 | 28.2850 | -25.4042 | -2.23 | -12.3 | 5.5 | -2.09 | -10.4 | 6.4 |
| HARDING | KWAZULU-NATAL | 42887 | 29.8835 | -30.5765 |  |  |  | -0.85 | 1.0 | 7.7 |
| HARRISMITH | FREE STATE | 53572 | 29.1276 | -28.2701 | 2.93 | 22.1 | -1.3 | -1.99 | -7.0 | 8.9 |
| HARTSWATER | NORTHERN CAPE | 91987 | 24.8091 | -27.7537 | -1.83 | -17.1 | -2.5 | -3.57 | -25.3 | 3.2 |
| HEIDELBERG (CAPE) | WESTERN CAPE | 68159 | 20.9564 | -34.0892 | -2.87 | -8.0 | 15.0 | -2.34 | -5.1 | 13.7 |
| HEILBRON | FREE STATE | 53645 | 27.9677 | -27.2860 | -3.03 | -19.1 | 5.2 | -1.50 | -8.8 | 3.2 |
| HERTZOGVILLE | FREE STATE | 92046 | 25.5042 | -28.1281 | -2.12 | -17.4 | -0.4 | -3.34 | -22.6 | 4.1 |
| HLUHLUWE | KWAZULU-NATAL | 43364 | 32.2709 | -28.0202 | -1.84 | -8.9 | 5.8 | -0.54 | 2.2 | 6.5 |
| HOBHOUSE | FREE STATE | 53853 | 27.1431 | -29.5292 |  |  |  | -2.26 | -17.9 | 0.2 |
| HOOPSTAD | FREE STATE | 92167 | 25.9050 | -27.8364 | -0.69 | -15.0 | -9.5 | -4.44 | -34.7 | 0.8 |
| HOPETOWN | NORTHERN CAPE | 92222 | 24.0827 | -29.6230 |  |  |  | -0.66 | -2.4 | 2.8 |
| HOTAZEL | NORTHERN CAPE | 92289 | 22.9616 | -27.2023 | -5.24 | -34.5 | 7.3 |  |  |  |
| HUMANSDORP | EASTERN CAPE | 69054 | 24.7700 | -34.0277 | -1.81 | -7.1 | 7.3 | -3.49 | -14.4 | 13.5 |
| IMPENDLE | KWAZULU-NATAL | 43565 | 29.8665 | -29.6001 | -4.14 | -16.2 | 16.9 | -3.47 | -11.3 | 16.4 |
| INGWAVUMA | KWAZULU-NATAL | 43687 | 31.9971 | -27.1334 | 1.97 | 15.5 | -0.2 | 1.70 | 14.7 | 1.1 |
| IXOPO | KWAZULU-NATAL | 43847 | 30.0592 | -30.1526 | -0.70 | 0.3 | 5.9 | 0.17 | 5.6 | 4.3 |
| JACOBSDAL | FREE STATE | 92343 | 24.7746 | -29.1277 | -0.17 | -4.5 | -3.2 | -0.11 | 0.4 | 1.4 |
| JAMESTOWN | EASTERN CAPE | 69408 | 26.8082 | -31.1223 | 4.99 | 25.3 | -14.6 | 2.04 | 9.3 | -7.0 |
| JAN KEMPDORP | NORTHERN CAPE | 92407 | 24.8376 | -27.9179 | -1.41 | -14.7 | -3.4 | -3.93 | -26.2 | 5.2 |
| JANSENVILLE | EASTERN CAPE | 69457 | 24.6667 | -32.9456 | 7.95 | 41.2 | -22.4 | -4.22 | -26.5 | 7.3 |
| JOUBERTINA | EASTERN CAPE | 69552 | 23.8539 | -33.8253 | -5.70 | -24.7 | 20.9 | -3.69 | -14.5 | 15.0 |
| JUNO | LIMPOPO | 21106 | 29.0145 | -23.6377 |  |  |  | -3.42 | -18.0 | 9.4 |
| KAKAMAS | NORTHERN CAPE | 92464 | 20.6232 | -28.7747 | 2.52 | 7.0 | -13.1 |  |  |  |
| KEI ROAD | EASTERN CAPE | 70003 | 27.5471 | -32.7027 |  |  |  | 1.02 | 11.6 | 3.4 |
| KEIMOES | NORTHERN CAPE | 92704 | 20.9702 | -28.7079 |  |  |  | 0.45 | 1.6 | -2.0 |
| KENHARDT | NORTHERN CAPE | 92761 | 21.1533 | -29.3474 | 0.72 | 2.3 | -3.5 | -0.09 | 0.1 | 0.8 |
| KENTON ON SEA | EASTERN CAPE | 70254 | 26.6729 | -33.6838 | -1.92 | -6.9 | 8.5 |  |  |  |
| KIBLER PARK | GAUTENG | 21742 | 28.0071 | -26.3251 | -3.09 | -20.9 | 3.8 | -1.91 | -8.5 | 6.7 |
| KING WILLIAM'S TOWN | EASTERN CAPE | 70455 | 27.3893 | -32.8790 | -1.96 | -2.7 | 13.0 |  |  |  |
| KINROSS | MPUMALANGA | 21927 | 29.0992 | -26.4210 | -2.79 | -18.8 | 3.5 | -1.57 | -9.1 | 3.5 |
| KIRKWOOD | EASTERN CAPE | 70501 | 25.4436 | -33.3999 | -0.08 | -1.0 | -0.4 | -1.73 | -10.5 | 3.4 |
| KLEINMOND | WESTERN CAPE | 70808 | 19.0313 | -34.3406 | -2.93 | -11.1 | 12.3 | -2.58 | -10.3 | 10.3 |
| KLEINSEE | NORTHERN CAPE | 70904 | 17.0672 | -29.6802 | 2.08 | 8.0 | -8.7 | 0.26 | -2.4 | -4.5 |
| KOMATIPOORT | MPUMALANGA | 22621 | 31.9615 | -25.4376 |  |  |  | -1.00 | -1.6 | 6.4 |
| KOMGA | EASTERN CAPE | 71408 | 27.8925 | -32.5762 | -2.28 | -8.7 | 9.5 | -2.33 | -8.5 | 10.2 |
| KROONDAL | NORTH WEST | 22928 | 27.3024 | -25.7173 | -2.16 | -13.4 | 3.9 | -2.54 | -13.5 | 6.8 |
| KWAMBONAMBI | KWAZULU-NATAL | 44324 | 32.0844 | -28.6022 | -1.29 | -0.9 | 9.4 | -3.93 | -18.4 | 13.1 |
| LADISMITH | WESTERN CAPE | 72071 | 21.2683 | -33.4940 | -7.25 | -38.9 | 19.1 | -2.18 | -13.8 | 3.7 |
| LADYBRAND | FREE STATE | 54839 | 27.4566 | -29.1970 |  |  |  | -1.63 | -3.7 | 9.4 |
| LAINGSBURG | WESTERN CAPE | 72257 | 20.8590 | -33.1971 | -5.19 | -30.9 | 10.6 | -5.36 | -30.7 | 12.1 |
| LAMBERTSBAAI | WESTERN CAPE | 72378 | 18.3052 | -32.0932 | -3.25 | -14.0 | 12.0 | -3.08 | -13.5 | 11.2 |
| LEPHALALE ELLISRAS | LIMPOPO | 16588 | 27.7370 | -23.6765 |  |  |  | -1.22 | -7.5 | 2.2 |
| LERATO | NORTH WEST | 23663 | 25.9832 | -25.4925 | -4.68 | -26.5 | 10.9 | -4.34 | -25.8 | 8.9 |
| LETABA | LIMPOPO | 23823 | 30.2600 | -23.8950 | -2.57 | -10.2 | 10.3 | -2.62 | -11.9 | 9.1 |
| LINDLEY | FREE STATE | 55043 | 27.9217 | -27.8812 | 0.66 | -3.7 | -9.0 | -0.26 | -4.3 | -2.2 |
| LOERIESFONTEIN | NORTHERN CAPE | 73217 | 19.4427 | -30.9514 | -3.20 | -19.8 | 5.8 |  |  |  |
| LUTZVILLE | WESTERN CAPE | 73513 | 18.3446 | -31.5591 | -0.65 | -3.9 | 1.2 | -0.39 | -3.5 | -0.4 |
| LYDENBURG | MPUMALANGA | 24576 | 30.4528 | -25.0949 | -3.62 | -17.7 | 11.3 | -3.01 | -16.8 | 7.3 |
| MABATLANE VAALWATER | LIMPOPO | 37425 | 28.1103 | -24.2954 | -4.26 | -24.0 | 10.1 | -4.15 | -23.2 | 10.0 |
| MABESKRAAL | NORTH WEST | 24832 | 26.8016 | -25.1995 | -0.41 | -6.2 | -3.0 | -2.83 | -25.2 | -2.6 |
| MACHADODORP | MPUMALANGA | 24873 | 30.2503 | -25.6636 | -2.04 | -5.5 | 10.8 | -2.10 | -7.4 | 9.4 |
| MACLEAR | EASTERN CAPE | 73635 | 28.3474 | -31.0684 |  |  |  | -3.30 | -12.8 | 13.6 |
| MADADENI | KWAZULU-NATAL | 44743 | 30.0350 | -27.7476 | 0.36 | 4.9 | 2.0 | -1.35 | -5.6 | 5.2 |
| MAFIKENG | NORTH WEST | 73692 | 25.6423 | -25.8643 | -4.81 | -27.4 | 11.1 |  |  |  |
| MAGADLA | EASTERN CAPE | 84957 | 28.6812 | -30.3390 | -4.82 | -26.4 | 12.2 |  |  |  |
| MAHLABATINI | KWAZULU-NATAL | 44768 | 31.4687 | -28.2343 | -0.84 | 0.7 | 7.5 | -2.13 | -7.5 | 9.5 |
| MAKHADO LOUIS TRICHARDT | LIMPOPO | 24375 | 29.9071 | -23.0412 |  |  |  | 0.34 | 3.9 | 1.2 |
| MAPUMULO | KWAZULU-NATAL | 44969 | 31.0666 | -29.1560 | -1.21 | -1.4 | 8.3 | -2.77 | -8.0 | 14.1 |
| MARBLE HALL | MPUMALANGA | 25376 | 29.2945 | -24.9692 | -0.05 | -0.9 | -0.5 |  |  |  |
| MARISHANE | LIMPOPO | 34697 | 29.7476 | -24.7276 | -2.86 | -14.9 | 8.0 |  |  |  |
| MARYDALE | NORTHERN CAPE | 93721 | 22.1048 | -29.4059 | -5.60 | -36.8 | 8.0 | -5.48 | -35.9 | 7.9 |
| MATATIELE | EASTERN CAPE | 74057 | 28.8101 | -30.3402 | -2.85 | -16.5 | 6.3 | -3.41 | -18.6 | 8.7 |
| MATSULU | MPUMALANGA | 25753 | 31.3540 | -25.5186 | -1.88 | -7.4 | 7.7 | -2.82 | -10.7 | 11.9 |
| MEER EN SEE | KWAZULU-NATAL | 45317 | 32.1014 | -28.7818 | 0.85 | 10.6 | 3.8 | -0.80 | -0.9 | 5.5 |
| MELMOTH | KWAZULU-NATAL | 45246 | 31.4011 | -28.5926 | -1.07 | -1.8 | 6.7 | -2.69 | -8.4 | 13.2 |
| MHLUZI | MPUMALANGA | 26216 | 29.4280 | -25.7601 | 1.40 | 8.5 | -2.7 | 0.74 | 3.5 | -2.5 |
| MIER | NORTHERN CAPE | 93762 | 20.0268 | -26.7443 | -3.79 | -27.8 | 2.6 | -3.84 | -27.4 | 3.3 |
| MKHUHLU | MPUMALANGA | 26433 | 31.2447 | -24.9862 | -2.74 | -10.7 | 11.2 | -3.32 | -13.3 | 13.2 |
| MODJADJI | LIMPOPO | 26789 | 30.3462 | -23.6297 | -3.18 | -12.4 | 13.0 |  |  |  |
| MOKOPANE POTGIETERSRUS | LIMPOPO | 30475 | 29.0109 | -24.1835 | 1.56 | 6.0 | -6.5 | 2.06 | 8.9 | -7.6 |
| MOOKETSI | LIMPOPO | 26827 | 30.0990 | -23.5935 |  |  |  | -2.92 | -16.1 | 7.2 |
| MOOKGOPHONG NABOOMSPRUIT | LIMPOPO | 27426 | 28.7122 | -24.5191 | -1.35 | -9.9 | 0.9 | -2.58 | -14.3 | 6.3 |
| MOORREESBURG | WESTERN CAPE | 75611 | 18.6631 | -33.1562 | -0.94 | -1.5 | 6.0 | 0.75 | 4.1 | -1.9 |
| MOREBENG SOEKMEKAAR | LIMPOPO | 34671 | 29.9279 | -23.4944 | -4.98 | -27.3 | 12.6 |  |  |  |
| MOROKWENG | NORTH WEST | 93875 | 23.7706 | -26.1237 | -3.35 | -22.5 | 4.3 | -3.44 | -23.1 | 4.4 |
| MOTETEMA | LIMPOPO | 27160 | 29.4637 | -25.0992 | 0.41 | 1.9 | -1.3 |  |  |  |
| MOTHIBISTAT | NORTHERN CAPE | 93859 | 23.4876 | -27.3987 | -4.53 | -28.5 | 7.8 |  |  |  |
| MOTSWEDI | NORTH WEST | 27194 | 25.8894 | -25.2841 | -3.44 | -24.7 | 2.9 | -6.22 | -38.1 | 11.6 |
| MOUNT AYLIFF | EASTERN CAPE | 75918 | 29.3680 | -30.8004 | -3.65 | -15.3 | 14.0 | -2.47 | -10.7 | 9.0 |
| MOUNT FRERE | EASTERN CAPE | 76035 | 28.9933 | -30.9014 |  |  |  | -2.38 | -7.8 | 11.2 |
| MT FLETCHER | EASTERN CAPE | 75975 | 28.5049 | -30.6912 | -3.41 | -14.7 | 12.6 |  |  |  |
| MULIMA | LIMPOPO | 27346 | 30.0365 | -23.3185 | -4.97 | -26.2 | 13.6 | -5.17 | -27.2 | 14.1 |
| MUSINA MESSINA | LIMPOPO | 26022 | 30.0367 | -22.3500 | -1.66 | -10.7 | 2.6 | -3.00 | -19.5 | 4.5 |
| MUTALE | LIMPOPO | 36989 | 30.5237 | -22.7371 | -2.22 | -10.5 | 7.2 | -3.52 | -14.8 | 13.4 |
| NDWEDWE | KWAZULU-NATAL | 46206 | 30.9376 | -29.5118 |  |  |  | -1.64 | -2.2 | 11.0 |
| NIEUWOUDTVILLE | NORTHERN CAPE | 77116 | 19.1131 | -31.3778 | -4.17 | -20.1 | 13.3 | -4.50 | -20.3 | 15.7 |
| NKANDLA | KWAZULU-NATAL | 46407 | 31.0904 | -28.6203 | -0.99 | 2.9 | 10.8 | -1.86 | -5.2 | 9.7 |
| NONGOMA | KWAZULU-NATAL | 46528 | 31.6465 | -27.9076 | -0.57 | -1.8 | 2.7 | -0.94 | -1.9 | 5.7 |
| NOTTINGHAM ROAD | KWAZULU-NATAL | 46640 | 29.9933 | -29.3575 | -0.36 | 3.6 | 6.5 |  |  |  |
| NOUPOORT | NORTHERN CAPE | 77470 | 24.9528 | -31.1802 | -3.74 | -21.4 | 8.5 | -3.70 | -22.6 | 7.0 |
| NQAMAKWE | EASTERN CAPE | 77534 | 27.9372 | -32.2027 | -5.31 | -19.8 | 22.6 | -2.64 | -4.3 | 16.8 |
| NQUTU | KWAZULU-NATAL | 46720 | 30.6765 | -28.2133 | -0.75 | -4.1 | 1.9 |  |  |  |
| NTLAZA | EASTERN CAPE | 77575 | 29.1075 | -31.5668 |  |  |  | -1.36 | 5.2 | 16.1 |
| NZHELELE | LIMPOPO | 28555 | 30.1847 | -22.8998 | -4.12 | -22.2 | 10.8 | -4.01 | -22.2 | 9.9 |
| OLIFANTSHOEK | NORTHERN CAPE | 94089 | 22.7366 | -27.9433 | -4.37 | -29.9 | 5.1 | -4.68 | -30.8 | 6.6 |
| ONRUSRIVIER | WESTERN CAPE | 78011 | 19.1766 | -34.4112 | -3.19 | -13.6 | 11.9 | -3.12 | -12.9 | 12.0 |
| ORANJEKRUIN | NORTHERN CAPE | 94240 | 21.2264 | -28.4626 | 0.60 | 0.1 | -4.6 |  |  |  |
| ORANJEVILLE | FREE STATE | 55951 | 28.2121 | -26.9945 | -2.37 | -14.6 | 4.3 | -6.05 | -40.8 | 7.6 |
| OSHOEK BORDER POST | MPUMALANGA | 75188 | 30.9877 | -26.2136 | -3.81 | -16.3 | 14.1 |  |  |  |
| PACALTSDORP | WESTERN CAPE | 78406 | 22.4586 | -34.0166 | -3.34 | -9.2 | 17.5 | -1.57 | -0.3 | 12.2 |
| PETRUS STEYN | FREE STATE | 56300 | 28.1294 | -27.6474 | -1.74 | -8.9 | 5.0 |  |  |  |
| PETRUSBURG | FREE STATE | 56239 | 25.4130 | -29.1148 | -4.61 | -29.6 | 7.3 | -4.79 | -30.8 | 7.5 |
| PETRUSVILLE | NORTHERN CAPE | 94441 | 24.6588 | -30.0820 |  |  |  | -0.63 | -3.0 | 2.0 |
| PHILIPPOLIS | FREE STATE | 56375 | 25.2742 | -30.2645 | -2.55 | -14.1 | 6.3 | -3.83 | -25.4 | 5.2 |
| PHUTHADITJHABA | FREE STATE | 56422 | 28.8178 | -28.5212 | -2.70 | -12.6 | 9.0 | -3.63 | -18.6 | 10.5 |
| PIET RETIEF | MPUMALANGA | 29926 | 30.8016 | -27.0046 | 3.61 | 21.8 | -7.1 | 0.84 | 6.7 | 0.0 |
| PLETTENBERGBAAI | WESTERN CAPE | 79214 | 23.3721 | -34.0572 | -4.10 | -17.7 | 15.1 | -1.66 | -5.2 | 8.1 |
| POLOKWANE PIETERSBURG | LIMPOPO | 29879 | 29.4534 | -23.9120 | -1.42 | -6.4 | 4.9 | -2.46 | -10.7 | 9.0 |
| POMEROY | KWAZULU-NATAL | 47328 | 30.4244 | -28.5519 | -3.33 | -14.8 | 11.9 |  |  |  |
| PONGOLA | KWAZULU-NATAL | 47369 | 31.6159 | -27.3778 | -0.79 | -1.0 | 5.4 | -1.63 | -5.1 | 8.0 |
| PORT EDWARD | KWAZULU-NATAL | 47408 | 30.2266 | -31.0518 | -1.95 | -4.7 | 10.9 | -2.64 | -7.2 | 13.9 |
| PORT NOLLOTH | NORTHERN CAPE | 79577 | 16.8700 | -29.2551 | 3.36 | 13.3 | -13.6 | 1.23 | 2.5 | -7.3 |
| PORT ST JOHNS | EASTERN CAPE | 79632 | 29.5403 | -31.6233 |  |  |  | -4.00 | -19.3 | 12.7 |
| POSTMASBURG | NORTHERN CAPE | 94801 | 23.0625 | -28.3326 | -4.44 | -30.4 | 5.1 | -4.61 | -30.8 | 6.0 |
| PRIESKA | NORTHERN CAPE | 94868 | 22.7499 | -27.6676 | -0.78 | -5.1 | 1.1 | -0.53 | -4.2 | 0.0 |
| PRINCE ALBERT | FREE STATE | 55399 | 29.5642 | -27.6815 | -5.60 | -30.6 | 14.2 |  |  |  |
| PRINCE ALBERT | WESTERN CAPE | 79699 | 22.0311 | -33.2275 | -6.12 | -33.2 | 15.8 | -5.62 | -31.3 | 13.6 |
| PRINCE ALFRED HAMLET | KWAZULU-NATAL | 44001 | 32.0687 | -27.4295 |  |  |  | 1.50 | 13.5 | 1.5 |
| PRINCE ALFRED HAMLET | WESTERN CAPE | 78270 | 19.3273 | -33.2930 | -5.36 | -27.3 | 15.7 |  |  |  |
| QAMATA | EASTERN CAPE | 79939 | 27.4344 | -31.9781 |  |  |  | -1.35 | 3.4 | 14.2 |
| RADITHUSO | NORTH WEST | 31227 | 25.6048 | -26.2002 | -4.90 | -29.1 | 10.1 | -5.09 | -30.5 | 10.2 |
| RADITSHABA | LIMPOPO | 31219 | 28.8456 | -22.8386 | -4.71 | -29.9 | 7.8 |  |  |  |
| RANKUNYANA | NORTH WEST | 93231 | 27.3038 | -25.5730 | -0.21 | -4.8 | -3.1 | -3.63 | -25.9 | 3.2 |
| REDDERSBURG | FREE STATE | 56519 | 26.1731 | -29.6523 | -3.31 | -21.0 | 5.5 | -3.14 | -17.1 | 8.0 |
| REITZ | FREE STATE | 56584 | 28.4297 | -27.8008 | -0.82 | -3.9 | 2.6 | -2.72 | -11.7 | 10.1 |
| REIVILO | NORTH WEST | 95049 | 24.1836 | -27.5673 |  |  |  | -2.45 | -15.3 | 4.3 |
| REYGERSDAL | WESTERN CAPE | 80630 | 18.4943 | -33.5634 | -2.07 | -8.6 | 7.9 | -0.85 | -0.4 | 6.4 |
| RHENOSTERKRAAL | NORTH WEST | 32000 | 26.9810 | -24.9969 | -3.72 | -21.0 | 8.7 | -4.23 | -25.3 | 8.5 |
| RICHMOND | MPUMALANGA | 18686 | 28.7490 | -26.7468 | -1.16 | -9.1 | 0.2 |  |  |  |
| RICHMOND (KZN) | KWAZULU-NATAL | 47641 | 30.2729 | -29.8717 | -0.31 | 3.5 | 5.9 | -0.69 | 2.9 | 8.4 |
| RICHMOND (N CAPE) | NORTHERN CAPE | 80831 | 23.9441 | -31.4140 | -3.48 | -20.2 | 7.6 | -3.31 | -19.3 | 7.2 |
| RIETKUIL | MPUMALANGA | 32197 | 29.8058 | -25.9503 | -0.86 | -1.7 | 5.1 | -1.30 | -3.7 | 6.7 |
| RIVERSDALE | WESTERN CAPE | 81134 | 21.2603 | -34.0921 | -4.51 | -17.6 | 18.5 | -1.22 | -0.7 | 9.0 |
| RIVERSDALE | NORTH WEST | 96188 | 24.7268 | -26.9602 | -3.15 | -10.5 | 14.7 |  |  |  |
| RIVIERSONDEREND | WESTERN CAPE | 80911 | 19.9125 | -34.1503 |  |  |  | -1.48 | -3.4 | 8.5 |
| ROOSSENEKAL | MPUMALANGA | 32775 | 29.9253 | -25.1948 | 1.09 | 4.2 | -4.5 | -2.17 | -9.2 | 8.2 |
| ROSSLYN | GAUTENG | 32870 | 28.0965 | -25.6255 | -2.64 | -17.7 | 3.4 | -1.50 | -8.2 | 3.8 |
| SABIE | MPUMALANGA | 33229 | 30.7800 | -25.0985 | -3.96 | -17.8 | 13.8 | -3.63 | -16.3 | 12.8 |
| SALDANHA | WESTERN CAPE | 82272 | 17.9444 | -33.0076 | -0.87 | -1.4 | 5.6 |  |  |  |
| SANTOY | NORTHERN CAPE | 95186 | 22.8355 | -27.1209 | -4.10 | -29.5 | 3.4 |  |  |  |
| SASOLBURG | FREE STATE | 56865 | 27.8295 | -26.8160 | -2.99 | -19.5 | 4.5 | -1.68 | -10.4 | 3.0 |
| SAULSPOORT | NORTH WEST | 33420 | 27.1672 | -25.1699 |  |  |  | -5.29 | -33.8 | 8.5 |
| SCHWEIZER-RENEKE | NORTH WEST | 95282 | 25.3300 | -27.1920 |  |  |  | -4.05 | -30.4 | 2.0 |
| SEBOKENG | GAUTENG | 33647 | 27.8389 | -26.5727 | -2.89 | -19.4 | 3.7 | -1.70 | -9.1 | 4.5 |
| SEDGEFIELD | WESTERN CAPE | 82818 | 22.8000 | -34.0145 | -3.69 | -11.0 | 18.5 | -3.65 | -11.3 | 17.9 |
| SELOSESHA | FREE STATE | 56946 | 26.8172 | -29.1957 | 2.14 | 10.7 | -6.4 |  |  |  |
| SENEKAL | FREE STATE | 56938 | 27.6234 | -28.3223 | 1.25 | 1.7 | -8.3 | -1.74 | -12.1 | 1.8 |
| SENWABARWANA BOCHUM | LIMPOPO | 12279 | 29.1394 | -23.2873 |  |  |  | -4.25 | -25.8 | 8.2 |
| SHONGWE MISSION | MPUMALANGA | 34212 | 31.5000 | -25.7009 |  |  |  | -1.67 | -4.4 | 8.9 |
| SIBASA | LIMPOPO | 34220 | 30.4721 | -22.9491 | -2.82 | -11.0 | 11.5 | -2.65 | -10.3 | 10.9 |
| SIQHOBONG | EASTERN CAPE | 82883 | 28.4188 | -30.4353 | -3.48 | -15.8 | 12.1 |  |  |  |
| SIYABUSWA | MPUMALANGA | 34462 | 29.0651 | -25.1108 | 6.69 | 35.6 | -18.0 |  |  |  |
| SLURRY | NORTH WEST | 34622 | 25.8460 | -25.8115 | -3.85 | -22.4 | 8.4 | -3.45 | -21.5 | 6.1 |
| SOMERSET EAST | NORTH WEST | 29121 | 26.0038 | -26.8116 |  |  |  | -2.41 | -9.7 | 9.6 |
| SOMERSET EAST | EASTERN CAPE | 83352 | 25.5850 | -32.7192 | -3.05 | -11.8 | 12.5 | -2.42 | -9.8 | 9.6 |
| SPRINGBOK | NORTHERN CAPE | 83771 | 17.8826 | -29.6647 | 2.38 | 8.9 | -10.1 | 0.43 | -1.5 | -4.9 |
| SPRINGFONTEIN | FREE STATE | 54695 | 27.2347 | -27.6619 | -0.78 | -5.6 | 0.6 | -3.39 | -20.9 | 6.2 |
| SPRINGFONTEIN | FREE STATE | 57289 | 25.7051 | -30.2660 | -0.48 | -4.6 | -0.8 | -3.15 | -21.5 | 3.7 |
| ST FRANCIS BAY | FREE STATE | 52388 | 27.6731 | -27.5549 | -3.23 | -17.3 | 8.5 | -1.48 | -4.8 | 7.0 |
| ST FRANCIS BAY | EASTERN CAPE | 81939 | 24.8271 | -34.1624 | -0.88 | -1.5 | 5.5 | -1.59 | -4.3 | 8.5 |
| STANDERTON | MPUMALANGA | 35174 | 29.2433 | -26.9537 | -1.20 | -6.7 | 2.9 | -2.25 | -11.4 | 6.6 |
| STEELPOORT | LIMPOPO | 35270 | 30.2060 | -24.7316 | -1.25 | -4.3 | 5.8 | -1.25 | -5.1 | 4.9 |
| STEINKOPF | NORTHERN CAPE | 84072 | 17.7347 | -29.2597 | 2.37 | 9.2 | -9.8 | 0.71 | -1.3 | -7.0 |
| STELLA | NORTH WEST | 95467 | 24.8674 | -26.5535 | -4.85 | -31.0 | 7.8 | -5.06 | -32.3 | 8.2 |
| STERKSPRUIT | FREE STATE | 50143 | 26.6441 | -27.7481 | -2.76 | -13.4 | 8.7 | -3.35 | -16.7 | 10.1 |
| STERKSPRUIT | EASTERN CAPE | 84194 | 27.3712 | -30.5277 | -2.83 | -14.2 | 8.4 | -3.28 | -17.0 | 9.3 |
| STERKSTROOM | EASTERN CAPE | 84258 | 26.5516 | -31.5553 | -4.29 | -23.4 | 10.9 | -4.35 | -24.0 | 10.7 |
| STEYTLERVILLE | EASTERN CAPE | 84434 | 24.3445 | -33.3284 | -2.44 | -12.0 | 7.5 |  |  |  |
| STILBAAI | WESTERN CAPE | 84505 | 21.4054 | -34.3693 |  |  |  | -4.11 | -18.6 | 14.3 |
| STRAND | WESTERN CAPE | 84798 | 18.8303 | -34.1163 | -1.67 | -4.6 | 8.8 | -0.31 | 1.6 | 4.1 |
| SUN CITY | NORTH WEST | 35664 | 27.0997 | -25.3615 | -0.87 | -8.3 | -1.4 | -5.29 | -28.6 | 13.7 |
| SUTHERLAND | NORTHERN CAPE | 85099 | 20.6602 | -32.3969 | -4.68 | -29.7 | 7.7 | -4.26 | -23.5 | 10.6 |
| SWARTRUGGENS | NORTH WEST | 35824 | 26.6892 | -25.6506 | 0.56 | 0.3 | -4.2 | -3.15 | -18.6 | 6.6 |
| SWELLENDAM | WESTERN CAPE | 85339 | 20.4436 | -34.0208 | -2.61 | -8.0 | 12.9 | -2.06 | -4.4 | 12.1 |
| TABANKULU | EASTERN CAPE | 85450 | 29.3025 | -30.9617 | -3.89 | -18.8 | 12.3 |  |  |  |
| TAMPOSTAD | NORTH WEST | 36005 | 26.4828 | -25.4685 | -5.59 | -33.3 | 11.4 |  |  |  |
| TAUNG STATION | NORTH WEST | 95707 | 24.7483 | -27.5611 | -0.63 | -12.2 | -7.2 |  |  |  |
| THABAZIMBI | LIMPOPO | 36223 | 27.4100 | -24.5901 | -2.82 | -16.1 | 6.5 | -2.70 | -15.2 | 6.4 |
| THEUNISSEN | FREE STATE | 57704 | 26.7114 | -28.4002 |  |  |  | -2.24 | -15.2 | 2.8 |
| THOHOYANDOU | LIMPOPO | 36344 | 30.4591 | -22.9752 | -0.07 | -0.9 | -0.3 |  |  |  |
| THULAMAHASHE | MPUMALANGA | 36298 | 31.1998 | -24.7241 | -0.81 | -2.1 | 4.4 | -2.64 | -8.9 | 12.3 |
| TIEGERPOORT | GAUTENG | 18838 | 28.3860 | -25.8919 |  |  |  | -1.81 | -9.2 | 5.3 |
| TLHAKGAMENG | NORTH WEST | 95772 | 24.3418 | -26.4690 | -4.69 | -32.3 | 5.2 | -5.78 | -34.9 | 11.4 |
| TOSCA | NORTH WEST | 95764 | 23.9595 | -25.8788 | -4.94 | -32.0 | 7.5 |  |  |  |
| TOUWSRIVIER | WESTERN CAPE | 86349 | 20.0315 | -33.3398 | -6.07 | -33.7 | 14.9 | -5.16 | -29.2 | 12.1 |
| TRICHARDTSDAL | LIMPOPO | 36826 | 30.4023 | -24.1696 | -4.47 | -23.0 | 12.7 |  |  |  |
| TROMPSBURG | FREE STATE | 57841 | 25.7796 | -30.0349 | -3.70 | -22.9 | 6.7 | -3.95 | -23.8 | 7.8 |
| TSOLO | EASTERN CAPE | 86478 | 28.7575 | -31.3099 | -3.93 | -18.3 | 13.2 |  |  |  |
| UITENHAGE | EASTERN CAPE | 86950 | 25.4048 | -33.7638 | -3.98 | -15.5 | 16.3 | -4.31 | -16.7 | 17.8 |
| UMBUMBULU | KWAZULU-NATAL | 48763 | 30.7033 | -29.9869 | -0.47 | 3.1 | 6.8 | -0.38 | 3.0 | 6.1 |
| UMHLALI | KWAZULU-NATAL | 48843 | 31.2171 | -29.4782 | -1.64 | -2.6 | 10.5 | -1.79 | -2.3 | 12.1 |
| UMZINTO | KWAZULU-NATAL | 49049 | 30.6631 | -30.3115 | -2.57 | -7.4 | 13.1 | -1.69 | -1.4 | 12.1 |
| UNDERBERG | KWAZULU-NATAL | 47127 | 29.4930 | -29.7933 | -2.64 | -9.7 | 11.4 | -2.63 | -7.4 | 13.7 |
| UNIONDALE | WESTERN CAPE | 87131 | 23.1267 | -33.6562 | -5.50 | -30.2 | 13.8 | -4.28 | -24.3 | 9.9 |
| UTRECHT | KWAZULU-NATAL | 49209 | 30.3218 | -27.6559 |  |  |  | -1.72 | -6.5 | 7.2 |
| VANDERKLOOF | NORTHERN CAPE | 95932 | 24.7418 | -29.9982 | -1.63 | -8.9 | 4.1 |  |  |  |
| VANWYKSVLEI | NORTHERN CAPE | 87438 | 21.8247 | -30.3503 | -3.73 | -28.3 | 1.6 |  |  |  |
| VANWYKSVLEI | NORTHERN CAPE | 87438 | 21.8247 | -30.3503 | -3.84 | -28.7 | 1.9 | -3.76 | -26.0 | 4.1 |
| VENTERSDORP | NORTH WEST | 37923 | 26.8234 | -26.3191 | -3.85 | -25.0 | 5.8 | -4.46 | -25.2 | 10.4 |
| VICTORIA WEST | NORTHERN CAPE | 87613 | 23.1108 | -31.4040 | -3.58 | -24.8 | 3.8 | -3.06 | -20.9 | 3.6 |
| VILLIERSDORP | WESTERN CAPE | 87735 | 19.2914 | -33.9908 | -3.34 | -19.3 | 7.4 | -2.90 | -17.3 | 5.9 |
| VLAKDRIF | NORTH WEST | 38320 | 27.4397 | -25.9938 | -4.83 | -23.1 | 15.5 | -4.31 | -23.9 | 10.6 |
| VOLKSRUST | MPUMALANGA | 38379 | 29.8832 | -27.3691 | 2.08 | 7.3 | -9.3 | -4.19 | -26.1 | 7.4 |
| VREDE | FREE STATE | 58754 | 29.1630 | -27.4269 |  |  |  | -5.84 | -43.0 | 3.7 |
| VREDEFORT | FREE STATE | 58826 | 27.3677 | -27.0063 | -0.80 | -4.9 | 1.5 | -1.26 | -6.8 | 3.3 |
| VREDENBURG | WESTERN CAPE | 88279 | 17.9875 | -32.9074 | -1.25 | -1.5 | 8.5 | 0.26 | 2.7 | 0.6 |
| VREDENDAL | WESTERN CAPE | 88333 | 18.5084 | -31.6641 | -1.50 | -7.5 | 4.5 | -0.90 | -6.4 | 0.9 |
| VRYHEID | KWAZULU-NATAL | 49400 | 30.7948 | -27.7675 | 0.18 | 3.7 | 2.3 | -3.10 | -11.6 | 13.2 |
| WARDEN | FREE STATE | 58891 | 28.9637 | -27.8559 | 4.09 | 19.0 | -13.8 | -2.23 | -13.2 | 4.6 |
| WARRENTON | NORTHERN CAPE | 96243 | 24.8481 | -28.1118 | -2.15 | -18.6 | -1.4 | -3.67 | -26.2 | 3.2 |
| WASBANK | KWAZULU-NATAL | 49521 | 30.1014 | -28.3116 | 0.69 | 4.8 | -0.7 | -2.05 | -8.4 | 8.0 |
| WEPENER | FREE STATE | 59177 | 27.0390 | -29.7289 | 0.01 | 1.7 | 1.7 | -3.07 | -17.5 | 7.1 |
| WESTVILLE | KWAZULU-NATAL | 49723 | 30.9177 | -29.8379 | 0.12 | 7.0 | 6.1 | -0.12 | 5.3 | 6.3 |
| WHITTLESEA | EASTERN CAPE | 88697 | 26.8252 | -32.1764 | -2.88 | -13.2 | 9.8 | -2.16 | -9.9 | 7.4 |
| WILLISTON | NORTHERN CAPE | 88815 | 20.9175 | -31.3407 | -4.42 | -30.4 | 4.9 | -4.59 | -30.7 | 6.0 |
| WILLOWMORE | EASTERN CAPE | 88872 | 23.4879 | -33.2959 | -7.26 | -41.4 | 16.6 | -6.75 | -39.6 | 14.4 |
| WINBURG | FREE STATE | 59667 | 27.0117 | -28.5196 | 3.28 | 11.4 | -14.9 | -1.91 | -16.5 | -1.1 |
| WINTERTON | KWAZULU-NATAL | 49844 | 29.5381 | -28.8136 |  |  |  | -3.06 | -9.4 | 15.0 |
| WOLMARANSSTAD | NORTH WEST | 39693 | 25.9830 | -27.1990 | -0.85 | -5.5 | 1.3 | -1.27 | -8.0 | 2.2 |
| ZASTRON | FREE STATE | 59949 | 27.0824 | -30.3035 | 1.80 | 7.6 | -6.8 | 0.69 | 2.6 | -2.9 |
